# The Influence of Face Masks on Micro-Expression Recognition

**DOI:** 10.3390/bs15020200

**Published:** 2025-02-13

**Authors:** Yunqiu Zhang, Chuanlin Zhu

**Affiliations:** School of Educational Science, Yangzhou University, Yangzhou 225002, China; mx120220232@stu.yzu.edu.cn

**Keywords:** micro-expression recognition, background emotions, masks, favorability

## Abstract

This study aimed to explore the influence of various mask attributes on the recognition of micro-expressions (happy, neutral, and fear) and facial favorability under different background emotional conditions (happy, neutral, and fear). The participants were asked to complete an ME (micro-expression) recognition task, and the corresponding accuracy (ACC), reaction time (RT), and facial favorability were analyzed. Results: (1) Background emotions significantly impacted the RT and ACC in micro-expression recognition, with fear backgrounds hindering performance. (2) Mask wearing, particularly opaque ones, prolonged the RT but had little effect on the ACC. Transparent masks and non-patterned masks increased facial favorability. (3) There was a significant interaction between background emotions and mask attributes; negative backgrounds amplified the negative effects of masks on recognition speed and favorability, while positive backgrounds mitigated these effects. This study provides insights into how masks influence micro-expression recognition, crucial for future research in this area.

## 1. Introduction

The study of micro-expressions can be traced back to Darwin’s publication of “The Expression of the Emotions in Man and Animals” in 1872. Subsequently, Haggard and Ekman discovered and defined the existence of micro-expressions (Haggard & Isaacs, 1966; Ekman & Friesen, 1969). Micro-expressions refer to brief facial expression changes that last between 40 and 200 ms, and Ekman noted that they typically occur when individuals attempt to suppress or conceal their true emotions (Ekman, 2002). These fleeting expressions reveal an individual’s genuine inner emotions and thus hold significant importance in psychological research. Friesen developed the first standard test for micro-expression recognition, the BART (Brief Affect Recognition Test) (Ekman & Friesen, 1974). The BART, created by Ekman and Friesen, was the first standard test for micro-expression recognition, assessing recognition abilities through the rapid presentation of facial expression images. This test is of great significance for understanding human nonverbal emotional communication. The BART aims to evaluate an individual’s ability to recognize micro-expressions by quickly presenting a series of images containing micro-expressions. The images used in the test typically include various types of micro-expressions, such as anger, fear, sadness, disgust, surprise, and happiness. Participants are required to watch and identify the rapidly flashed facial expression images on the screen. Due to the extremely short duration of micro-expressions, this demands a high level of attention and rapid recognition ability from participants. Test results are usually measured by the number of correctly identified expressions or recognition ACC. Building on this, Ekman and Friesen established the Facial Action Coding System (FACS) (Ekman & Friesen, 1975), providing a systematic methodological foundation for micro-expression research(Ekman & Friesen, 1978).Currently, multiple micro-expression databases have been constructed domestically and internationally, providing rich materials and data support for subsequent research. Subsequently, Matsumoto developed the JACBART (Japanese and Caucasian Brief Affect Recognition Test) (Matsumoto et al., 2000). The JACBART improved upon the BART in two main aspects: firstly, it included faces of Japanese and Caucasian individuals, making the test more cross-culturally applicable; secondly, it not only assessed basic emotion recognition abilities but also added an examination of micro-expression recognition abilities, providing a more comprehensive assessment of emotional recognition abilities. These classic tests laid the foundation for micro-expression research, but they were mainly limited to neutral faces as background expressions, which had low ecological validity and could not fully reflect emotional expressions in the real world. Zhang pointed out that micro-expressions in real environments usually accompany various dynamic expressions, rather than appearing only on neutral faces (J. Zhang et al., 2017). To enhance the ecological validity of research, they created the EMERT (Ecological Microexpressions Recognition Test), examining the recognition of six micro-expressions (happiness, anger, sadness, fear, surprise, and disgust) against a backdrop of seven basic expressions (happiness, anger, sadness, fear, surprise, disgust, and contempt). The results found significant background effects, supporting the necessity of an ecological research paradigm. By providing a more realistic micro-expression recognition context, the EMERT improves the external validity of experimental results, helping to more accurately reflect the manifestation of micro-expressions in real life. Furthermore, the application of the EMERT in different populations has shown that it can help us better understand the differences in micro-expression recognition between clinical and healthy individuals. For example, one study compared the performances of individuals with depression and healthy individuals in ecological micro-expression recognition and found that background expressions significantly affected the ACC and RT of micro-expression recognition; another study explored the characteristics of ecological micro-expression recognition in young people with subclinical depression, and the results indicated that background expressions significantly affected recognition performance, and there were biases in the misjudgment of micro-expressions under different situations. These studies emphasize the importance and advantages of using ecological micro-expression recognition tests in practical applications.

Meanwhile, a plethora of research has explored the factors influencing micro-expression recognition. Frank found that the activation levels of higher-order face processing areas, such as the Fusiform Face Area (FFA) and the Superior Temporal Sulcus, are positively correlated with participants’ abilities to recognize micro-expressions. Shen et al. demonstrated that individuals with stronger Theory of Mind (ToM) capabilities have higher ACC in recognizing micro-expressions (W. Shen et al., 2016). Additionally, cognitive factors such as attentional breadth and working memory capacity have been proven to be related to micro-expression recognition performance. Moreover, the presence of contextual cue information significantly affects the recognition of micro-expressions. For instance, Shen et al. discovered that briefly presented background emotional words can influence facial expression recognition (X. Shen et al., 2015). In their study, emotional words were presented before the micro-expressions, with participants first seeing the briefly presented emotional words, followed immediately by the facial micro-expressions. This sequence allowed the emotional words to serve as contextual cues affecting participants’ recognition and judgment of subsequent micro-expressions. The results indicated that emotional words could guide participants’ attention, thereby significantly influencing their recognition performance of facial micro-expressions. Zhang et al. revealed the background valence effect in micro-expression recognition, showing that different emotional backgrounds can affect the recognition of micro-expressions (M. Zhang et al., 2018). In their experiment, participants were asked to recognize micro-expressions presented against various emotional backgrounds. The experimental procedure was as follows: first, participants watched a video clip presenting an emotional background, such as happiness, sadness, anger, or fear, and then a micro-expression was briefly presented within this emotional context. The results showed that when the micro-expression was consistent with the background emotion, participants’ recognition ACC significantly improved; conversely, when the micro-expression was inconsistent with the background emotion, recognition ACC significantly decreased. This suggests that background valence has a significant impact on micro-expression recognition, supporting the existence of the background valence effect. The background valence effect is characterized by the positive or negative valence of background emotions affecting the recognition of micro-expressions, with positive backgrounds aiding in the recognition of positive micro-expressions and negative backgrounds aiding in the recognition of negative micro-expressions. Subsequently, Zhang et al. further verified the impact of emotional context on micro-expression recognition (M. Zhang et al., 2018). Their experiment indicated that negative and positive contexts can occupy attentional resources, thereby affecting the processing of micro-expressions. Specifically, in the experiment, participants first saw an emotional background (e.g., anger, sadness, or neutral), followed by the presentation of a target micro-expression (e.g., fear, surprise). The experimental results showed that when the background expression and the target micro-expression conveyed different emotions, recognition ACC significantly decreased, and when the emotional valence of the two was consistent, recognition was better. This suggests that the consistency of emotional valence between the background expression and the target micro-expression significantly influences the recognition of micro-expressions.

In recent years, the widespread use of face masks has provided a new opportunity to study the impact of facial occlusion on facial expression processing. Previous research has mainly focused on macro-expressions, with less attention on micro-expressions. Macro-expressions refer to facial expressions that are longer in duration, higher in intensity, and easier to detect, such as anger, happiness, and sadness (Ben et al., 2021). Carbon found that wearing a face mask reduces the ACC of macro-expression recognition, but there is currently little research systematically examining the modulating effect of facial occlusion cues like face masks on micro-expression cognitive processing (Carbon, 2020). Micro-expressions are brief, subtle, and often unconscious expressions, and studying their recognition under conditions of facial occlusion will help to better understand the complexity of emotional expression. Grundmann et al. demonstrated that face masks reduced the average ACC of recognizing six basic emotions by 12% (Grundmann et al., 2021). In this study, the participants were required to complete an emotion recognition task online. The study procedure was as follows: The participants first saw a facial expression image displaying one of the six basic emotions (happiness, anger, sadness, surprise, disgust, and fear), with some images showing faces wearing face masks. Then, the participants needed to select the type of emotion displayed in the image.

The experimental results indicated that the ACC of facial expression recognition was significantly reduced when face masks were worn. Specifically, the impact of face masks varied across different emotion types. For instance, the influence of face masks was relatively minor for expressions conveying happiness and surprise, whereas it was more pronounced for expressions of anger and disgust. This suggests that the impact of facial occlusions, such as face masks, on emotion recognition is dependent on the emotion type. Kotsia et al. found that for expressions of disgust, wearing a face mask paradoxically enhanced recognition ACC (Kotsia et al., 2021). In this study, the researchers utilized deep learning algorithms to analyze the impact of face masks on expression recognition, specifically evaluating the ACC and RT. The findings demonstrated that wearing a face mask significantly improved the ACC of recognizing expressions of disgust, while generally decreasing the recognition ACC for other expressions. Furthermore, the negative impact of transparent face masks was less than that of opaque face masks, with transparent masks showing greater advantages in maintaining recognition ACC and reducing the RT.

Although wearing face masks has been shown to significantly impact the recognition of macro-expressions, their effect on micro-expression recognition remains unclear and requires further investigation to determine their specific effects. In light of the aforementioned issues, this study aims to build upon Zhang et al.’s EMERT (Ecological Microexpressions Recognition Test) paradigm by introducing facial cues such as face masks to systematically examine their modulating effects on micro-expression recognition against different background expressions. The EMERT paradigm is an improved micro-expression recognition test designed to enhance the ecological validity of the test. Unlike the classic JACBART (Japanese and Caucasian Brief Affect Recognition Test), the EMERT not only examines micro-expression recognition against a neutral expression background but also incorporates seven basic expressions (happiness, anger, sadness, fear, surprise, disgust, and contempt) as context. The specific experimental procedure is as follows: in each trial, participants first view a background expression image lasting 800 ms, followed by a rapid presentation of a target micro-expression within 133 ms, and then another 800 ms presentation of the background expression image. Through this design, the EMERT can more realistically simulate the process of micro-expression recognition in actual situations.

Micro-expressions, as a key avenue for insight into an individual’s psychology, play a significant role. Research in the field of psychology indicates that emotional expression is composed of three parts: tone (7%), voice (38%), and facial expression (55%), with the latter primarily involving movements of the facial organs and body posture. Therefore, micro-expressions hold significant importance in social interactions. Moreover, their potential applications in fields such as justice, clinical medicine, advertising and consumer behavior, criminal investigation, and education are widely recognized. Studying micro-expressions not only aids professionals in identifying suspicious or deceptive behaviors but also enhances understanding of others’ true psychological states in daily interpersonal interactions, improving communication skills. With the increased prevalence of face mask wearing in modern society, the psychological distance between people seems to have been further extended by this facial obstruction. If individuals could selectively wear different types of masks when necessary and if people could more rapidly and accurately recognize micro-expressions beneath the masks, this would alter the interpersonal phenomenon and promote social harmony.

This study comprehensively examines the impact of wearing face masks on micro-expression recognition through three experiments: Experiment 1 compares the effects of wearing a face mask versus not wearing one on the ACC, RT, and facial favorability of micro-expression recognition; Experiment 2 explores the differential impacts of transparent versus opaque face masks; Experiment 3 focuses on the influence of the presence or absence of patterns on the surface of face masks on micro-expression recognition. Through these experiments, we aim to systematically investigate the modulating effects of different types of face masks on micro-expression recognition under different background expressions and preliminarily explore their specific mechanisms.

## 2. Experiment 1: The Impact of Wearing a Face Mask on Micro-Expression Recognition

Experiment 1 employed a within-subjects design of 3 (background expressions: happy, neutral, fear) × 3 (micro-expressions: happy, neutral, fear) × 2 (mask wearing: yes, no) to investigate the effects of different types of background expressions and mask wearing on micro-expression recognition and facial favorability. Based on previous research, the main effect of background expressions on the recognition of fear, sadness, disgust, and anger micro-expressions is significant, while the main effect for surprise and happy micro-expressions is not significant, but there are extensive significant differences with ordinary expressions. The valence of the background expression (i.e., the positivity or negativity of the emotion) affects the recognition of micro-expressions. When the background expression is negative, the recognition ACC of the target micro-expression is lower than that under positive or neutral background expressions. This suggests that negative background expressions impair participants’ capture of target micro-expressions.

In some cases, when the valence of the background expression and the target micro-expression is consistent (both positive or both negative), the recognition ACC of micro-expressions is significantly lower than when they are inconsistent. This indicates that the consistency between the valence of background expressions and target micro-expressions may interfere with micro-expression recognition. Additionally, background expressions interact with micro-expressions, as mentioned in the text, with a significant interaction effect between background expressions and micro-expressions. This implies that there are differences in the recognition of various micro-expressions under the same background expression, and the recognition of the same micro-expression also varies under different background expressions.

For the four micro-expressions of fear, sadness, disgust, and anger, the main effect of the background is significant, indicating that the recognition of these micro-expressions is significantly influenced by background expressions. Pairwise comparisons show extensive differences in the recognition of micro-expressions under different backgrounds, indicating that background expressions affect the recognition of these four ecological micro-expressions (J. Zhang et al., 2017). Another study found that when observing the whole body, the ACC of facial expression recognition was not significantly affected by masks except for happy expressions. Specifically, for expressions of anger, sadness, and fear, recognition ACC did not significantly decrease even when the face was obscured by a mask. However, for the recognition of happy expressions, recognition ACC significantly decreased when the face was obscured by a mask (Carbon, 2020). Regarding the favorability of facial recognition when wearing a mask, researchers have explored the impact of mask wearing on the ACC of emotional recognition and social judgments (including perceived trustworthiness, favorability, and closeness). The results showed that wearing a mask reduced the ACC of emotional recognition, making the target person appear less approachable. Specifically, the ACC of emotional recognition for target faces not wearing masks was 69.9%, and this ACC decreased for target faces wearing masks. Additionally, the study found that wearing a mask could buffer the negative effects of negative emotional expressions (such as anger, sadness, fear, and disgust) on trustworthiness, favorability, and closeness. That is, when the target person expresses negative emotions, people wearing masks are perceived as more trustworthy, likable, and approachable than those not wearing masks. However, the study also pointed out that wearing a mask itself did not directly affect social judgments, but when controlling for the positivity of expressed emotions and associations related to masks, mask wearing predicted lower perceived closeness. Furthermore, the study found that the stronger the threat associations related to masks, the higher the perceived closeness to target persons wearing masks (Grundmann et al., 2021).

Therefore, this experiment proposes the following hypotheses:

**Hypothesis** **1:** *The emotional valence of the background expression will affect the ACC of micro-expression recognition. Specifically, the ACC of micro-expression recognition will be highest under a happy background and lowest under a fear background. This is because positive background emotions can enhance an individual’s emotional recognition abilities, while negative background emotions may increase the difficulty of recognition.*

**Hypothesis** **2:** *Wearing a face mask will significantly reduce the ACC of micro-expression recognition and prolong the RT. The face mask obscures parts of the face, potentially leading to the loss of micro-expression details, thereby reducing recognition ACC.*

**Hypothesis** **3:** *There is an interaction between background expressions and face mask wearing. Under a fear background, the negative impact of wearing a face mask on micro-expression recognition is particularly pronounced. The fear background increases the emotional burden on individuals, making the obstruction of facial expressions further exacerbate the difficulty of recognition.*

**Hypothesis** **4:** *The obstruction by face masks will affect the favorability of facial expressions. Wearing a face mask will decrease the favorability of recognizing happy and neutral micro-expressions, while increasing the favorability of recognizing fear micro-expressions.*

### 2.1. Participants

Based on G*Power 3.1 calculations with an effect size f = 0.25 and α = 0.05, a total sample size of 14 was needed. To ensure a sufficiently large sample size, this experiment ultimately recruited 40 participants, all of whom were college students (equal numbers of males and females, with an average age of 21.05 ± 2.34 years (M ± SD) in Experiment 1). All participants were right-handed, with normal or corrected-to-normal vision, and had no history of psychiatric or neurological disorders. All participants signed an informed consent form before the experiment, acknowledging the purpose and procedures of the experiment, and ensuring the anonymity and confidentiality of the data. This study was conducted according to the ethical principles of the Declaration of Helsinki and received approval from the Research Ethics Committee of the School of Education Science of Yangzhou University (JKY-2023061301).

### 2.2. Materials

Experimental materials were sourced from the NimStim Face Stimulus Set (Tottenham et al., 2009). The NimStim Face Stimulus Set is a standardized library of facial expression images used for the study of human emotional expressions. Tottenham and colleagues published both background and micro-expression images in the NimStim Face Stimulus Set.

Background expressions included three types of expressions (happy, neutral, fear), with 18 images for each, totaling 54 images. Each set of facial expression images consisted of an equal number of male and female faces, with 8 male and 10 female images for each expression. Micro-expression images also included three types of expressions (happy, neutral, fear), with 18 images for each, totaling 54 images. Each set of facial expression images consisted of an equal number of male and female faces, with 8 male and 10 female images for each expression.

All images were available in both masked and unmasked versions, resulting in a total of 54 background expression images and 54 micro-expression images for use in the experiment. E-prime 2.0 software was used to present the stimuli and record data.

### 2.3. Procedure

The experimental procedure consisted of two stages: a practice phase and a formal experimental phase. During the practice phase, the participants were provided with 18 practice trials to ensure that they fully understood the experimental procedures. Feedback was given for each trial in the training phase, whereas no feedback was provided during the formal experiment. The flowchart for the training phase was identical to that of the formal phase. Based on previous research, the formal phase comprised 6 blocks, with each block containing 60 trials, totaling 360 trials (Zhu et al., 2017). The participants were allowed a 3 min break between every two blocks. Throughout the experiment, the participants were first seated in front of a computer, adjusting their viewing distance to ensure that the distance between the participant and the screen was approximately 50 cm, guaranteeing visual comfort and the ACC of the experimental data.

**Figure 1:**
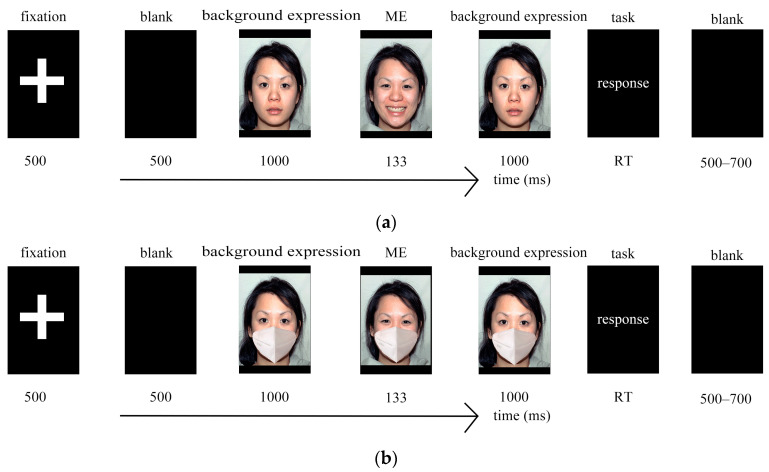
Flowchart of a single trial in Experiment 1 (**a**) illustrates the micro-expression recognition process without wearing a mask, while (**b**) shows the micro-expression recognition process with a mask on).

The experimental procedure for each trial was as follows: First, a 500 ms fixation point “+” was presented in the center of the screen to focus the participants’ attention. Subsequently, a background expression (happy, neutral, fear) was displayed for 1000 ms. Next, a micro-expression face image (happy, neutral, fear) was rapidly presented for 133 ms. Then, the same background expression reappeared for 1000 ms. The participants were then required to discern the micro-expression that was rapidly presented between the preceding and following background expressions and select the type of micro-expression they saw on the screen. For selection, keyboard buttons were used: “A” indicated happy, “F” indicated neutral, and “J” indicated fear. After each micro-expression recognition task, a judgment of facial favorability followed immediately. The participants were required to rate the favorability of the face on a 9-point scale ranging from 1 to 9, with higher numbers indicating greater favorability. The numbers 1 through 9 on the keyboard corresponded to the scale points 1 through 9, respectively. After the button selection, a blank screen of 500 to 700 ms was presented before proceeding to the next trial (Figure 1).

### 2.4. Data Analysis

Data analysis was conducted using SPSS 25.0 software.

The experimental data included the ACC and RT of micro-expression recognition, and facial favorability ratings. A 3 (background expressions: happy, neutral, fear) × 3 (micro-expressions: happy, neutral, fear) × 2 (mask wearing: yes, no) repeated measures analysis of variance (ANOVA) was conducted to examine the effects of background expressions, types of micro-expressions, and mask types on the ACC and RT of micro-expression recognition, and facial favorability ratings. Results that did not meet the assumption of sphericity were corrected using the Greenhouse–Geisser correction method. All post hoc tests employed the Bonferroni correction. Partial eta squared ($\eta_{p}^{2}$) was used to describe the effect size. The RTs were recorded from the appearance of the judgment interface to the moment the participant pressed the corresponding key.

### 2.5. Results and Discussion

#### 2.5.1. The ACC of Micro-Expression Recognition Task

Data analysis revealed a significant main effect of background expressions, with F(2,78) = 5.641, *p* = 0.009, and $\eta_{p}^{2}$ = 0.126. Compared with the fear background expression (0.82 ± 0.220), the participants had a higher ACC rate under the happy background expression condition (0.872 ± 0.183) (*p* = 0.020). Differences under other conditions were not significant (*p* > 0.05). This suggests that fear background expressions increase the difficulty of micro-expression recognition and decrease the ACC of recognition. A possible explanation is that fear background expressions belong to negative emotional stimuli, and individuals have lower precision in recognizing negative emotional categories.

The main effect of micro-expressions was not significant, with F(2,78) = 0.217, *p* = 0.806, and $\eta_{p}^{2}$ = 0.006. The main effect of face masks was not significant, with F(1,39) = 0.026, *p* = 0.872, and $\eta_{p}^{2}$ = 0.001. The interaction effect between background expressions and micro-expressions was not significant, with F(4,156) = 0.798, *p* = 0.462, and $\eta_{p}^{2}$ = 0.020; the interaction effect between background expressions and face masks was not significant, with F(2,78) = 0.959, *p* = 0.375, and $\eta_{p}^{2}$ = 0.024; and the interaction effect between micro-expressions and face masks was not significant, with F(2,78) = 2.725, *p* = 0.072, and $\eta_{p}^{2}$ = 0.065. The three-way interaction effect among background expressions, micro-expressions, and face masks was not significant, with F(4,156) = 1.874, *p* = 0.134, and $\eta_{p}^{2}$ = 0.046.

Through these data analysis results, it can be seen that background expressions influenced the ACC of micro-expression recognition. Compared to the happy background expression, the fear background particularly significantly reduced the ACC.

#### 2.5.2. The RT in the Micro-Expression Recognition Task

The main effect of micro-expressions was significant, with F(2,78) = 8.415, *p* < 0.001, and $\eta_{p}^{2}$ = 0.177. The post hoc test results indicated that compared with happy micro-expressions (993.10 ± 466.56), the participants had a longer RT for neutral micro-expressions (1164.53 ± 591.30) (*p* = 0.003); compared with happy micro-expressions (993.10 ± 466.56), the participants also had a longer RT for fear micro-expressions (1134.98 ± 616.48) (*p* = 0.015). Differences under other conditions were not significant (*p* > 0.05). The main effect of face masks was significant, with F(1,39) = 14.876, *p* < 0.001, and $\eta_{p}^{2}$ = 0.276. The post hoc test results indicated that the participants had a longer RT when wearing face masks (1154.43 ± 589.91) compared with not wearing face masks (1040.64 ± 526.31).

The interaction effect between background expressions and micro-expressions was significant, with F(4,156) = 7.982, *p* < 0.001, and $\eta_{p}^{2}$ = 0.041. The interaction effect between micro-expressions and face masks was significant, with F(2,78) = 3.702, *p* = 0.041, and $\eta_{p}^{2}$ = 0.087. The three-way interaction effect among background expressions, micro-expressions, and face masks was significant, with F(4,156) = 3.007, *p* = 0.020, and $\eta_{p}^{2}$ = 0.072.

Due to the significant interaction between background expressions and micro-expressions, simple effect analyses were conducted. The results are discussed below.

As can be seen from Table 1, the results of the first six groups were significant. These results indicate that individuals’ RTs in recognizing micro-expressions were influenced by the interaction between face masks and micro-expressions. The significant increase in the RT when wearing a mask while recognizing happy and neutral micro-expressions suggests that the mask increased the recognition duration for happy and neutral micro-expressions; however, the RTs for recognizing fear micro-expressions were not affected by the mask obstruction. Therefore, in the process of recognizing fear micro-expressions, the presence or absence of a mask did not significantly impact the RT, while the recognition of happy and neutral micro-expressions varied with the obstruction by the mask.

In summary, individuals’ RTs in recognizing micro-expressions were significantly affected by background expressions and micro-expressions (MEs), as well as the interaction between these two factors, regardless of whether a face mask was worn.

Due to the significant interaction between face masks and micro-expressions, simple effect analyses were conducted. The results are discussed below.

As can be seen from Table 2, the results of the first five groups were significant. These results indicate that individuals’ RTs in recognizing micro-expressions were influenced by the interaction between face masks and micro-expressions. The significant increase in the RT when wearing a mask while recognizing happy and neutral micro-expressions suggests that the mask increased the recognition duration for happy and neutral micro-expressions; however, the RTs for recognizing fear micro-expressions were not affected by the mask obstruction. Therefore, in the process of recognizing fear micro-expressions, the presence or absence of a mask did not significantly impact the RT, while the recognition of happy and neutral micro-expressions varied with the obstruction by the mask.

In summary, individuals’ RTs in recognizing micro-expressions under different background expressions were significantly affected by the presence of face masks and micro-expressions (MEs), as well as the interaction between these two factors.

The main effect of background expressions was not significant, with F(2,78) = 2.132, *p* = 0.130, and $\eta_{p}^{2}$ = 0.052; the interaction effect between background expressions and face masks was not significant, with F(2,78) = 1.679, *p* = 0.193, and $\eta_{p}^{2}$ = 0.041.

These data analysis results indicate the combined impact of background expressions, types of micro-expressions, and mask wearing on the RT in micro-expression recognition. The combination of a fear background and wearing a mask significantly prolonged the RT, while the impact of masks was less pronounced under happy and neutral backgrounds. These results provide an important theoretical basis for understanding the integrated effects of mask wearing and background context on emotion recognition.

#### 2.5.3. The Favorability of Micro-Expression Face Recognition

The main effect of background expressions was significant, with F(2,78) = 15.284, *p* < 0.001, and $\eta_{p}^{2}$ = 0.282. The post hoc test results indicated that compared with neutral background expressions (4.68 ± 0.92), the participants had higher favorability ratings for micro-expression recognition under happy background expressions (4.89 ± 1.06) (*p* = 0.013); compared with fear background expressions (4.43 ± 0.93), the participants had higher favorability ratings for micro-expression recognition under happy background expressions (4.89 ± 1.06) (*p* = 0.001); and differences under other conditions were not significant (*p* > 0.05). This suggests that happy background expressions enhance the favorability of micro-expression recognition, indicating that the presence of positive background expressions can influence the favorability of micro-expression recognition.

The main effect of micro-expressions was significant, with F(2,78) = 76.729, *p* < 0.001, and $\eta_{p}^{2}$ = 0.663. The post hoc test results indicated that compared with neutral micro-expressions (4.53 ± 0.76), the participants had higher favorability ratings for micro-expression recognition under happy micro-expressions (5.67 ± 1.20) (*p* < 0.001); compared with fear micro-expressions (3.79 ± 0.95), the participants had higher favorability ratings for micro-expression recognition under happy micro-expressions (5.67 ± 1.20) (*p* < 0.001); and compared with fear micro-expressions (3.79 ± 0.95), the participants had higher favorability ratings for micro-expression recognition under neutral micro-expressions (4.53 ± 0.76) (*p* < 0.001). This indicates that fear micro-expressions reduced the participants’ favorability ratings for micro-expression recognition.

The Interaction effect between background expressions and micro-expressions was significant, with F(4,156) = 15.853, *p* < 0.001, and $\eta_{p}^{2}$ = 0.289; the interaction effect between micro-expressions and face masks was significant, with F(2,78) = 30.882, *p* < 0.001, and $\eta_{p}^{2}$ = 0.442.

Due to the significant interaction between background expressions and micro-expressions, simple effect analyses were conducted. The results are discussed below.

As can be seen from Table 3, the results of the first nine groups were significant. These results indicate that individuals’ favorability ratings for micro-expression recognition were influenced by the interaction between background expressions and micro-expressions. Regardless of mask wearing, the favorability rating for recognizing happy micro-expressions was higher with a happy background expression compared with a neutral or fear background expression, and higher with a neutral background expression compared with a fear background expression. This suggests that happy and neutral background expressions enhanced the favorability of recognizing happy micro-expressions, while fear background expressions reduced the favorability. When not wearing a mask, recognizing neutral and fear micro-expressions was always reduced in favorability with a fear background expression, thus indicating that fear background expressions reduced the favorability of micro-expression recognition, while recognizing other micro-expressions varied with changes in background expressions.

In summary, individuals’ favorability ratings for micro-expression recognition, whether wearing a mask or not, were significantly influenced by background expressions and micro-expressions, as well as the interaction between these two factors.

Due to the significant interaction between face masks and micro-expressions, simple effect analyses were conducted. The results are discussed below.

As can be seen from Table 4, the results of the first four groups were significant. These results indicate that individuals’ favorability ratings for micro-expression recognition were influenced by the interaction between face masks and micro-expressions. Against happy and neutral background expressions, the favorability rating for recognizing happy micro-expressions was higher without a mask than with a mask; against a fear background expression, the favorability rating for recognizing neutral micro-expressions was higher without a mask than with a mask; and notably, against a fear background expression, the favorability rating for recognizing fear micro-expressions was higher with a mask than without a mask, suggesting that wearing a mask can reduce the negative impact of recognizing fear micro-expressions.

In summary, individuals’ favorability ratings for micro-expression recognition under different background expressions were significantly affected by the wearing of face masks and micro-expressions (MEs), as well as the interaction between these two factors.

The main effect of face masks was not significant, with F(1,39) = 3.107, *p* = 0.086, and $\eta_{p}^{2}$ = 0.074; the interaction effect between background expressions and face masks was not significant, with F(2,78) = 0.395, *p* = 0.675, and $\eta_{p}^{2}$ = 0.010. The three-way interaction effect among background expressions, micro-expressions, and face masks was not significant, with F(4,156) = 0.750, *p* = 0.535, and $\eta_{p}^{2}$ = 0.019.

The results of Experiment 1 indicate that wearing a mask significantly affected the reaction time (RT) of micro-expression recognition but had no significant effect on recognition accuracy (ACC). For the ACC of micro-expression recognition, background expressions had a significant effect. For the RT of micro-expression recognition, micro-expressions had a significant effect, background expressions had a significant effect, and significant interactions were observed between background expressions and micro-expressions, between micro-expressions and face masks, and among micro-expressions, background expressions, and face masks. The negative impact of wearing a mask on the RT was particularly evident under fear background expressions, increasing the RT for micro-expression recognition. This is in line with Hypothesis 3 of Experiment 1. Additionally, facial favorability ratings were the highest under happy background expressions and the lowest under fear background expressions, with favorability ratings generally being lower when masks were worn compared with when they were not. These results suggest that wearing a mask, especially under fear background expressions, negatively affects both micro-expression recognition and facial favorability, which is consistent with Hypothesis 4 of Experiment 1. The focus of Experiment 1 was to explore the impact of wearing face masks on the recognition of micro-expressions; therefore, does the degree of obstruction by the mask affect micro-expression recognition? Consequently, Experiment 2 of this study designed masks with varying levels of transparency to investigate their impact on the recognition of micro-expressions, as well as the effects of background expressions, micro-expressions, and masks of different transparency levels on micro-expression recognition and facial favorability.

## 3. Experiment 2: The Impact of Mask Transparency on Micro-Expression Recognition

Experiment 2 employed a within-subjects design of 3 (background expressions: happy, neutral, fear) × 3 (micro-expressions: happy, neutral, fear) × 2 (mask types: opaque white mask, transparent mask) to investigate the impact of different types of background expressions and mask wearing on micro-expression recognition and facial favorability. This experiment proposes the following hypotheses:

**Hypothesis** **5:** *The emotional valence of background expressions will affect the ACC of micro-expression recognition. Specifically, the ACC of micro-expression recognition will be highest under happy backgrounds and lowest under fear backgrounds. This is because positive background emotions can enhance an individual’s emotional recognition ability, while negative background emotions may increase the difficulty of recognition.*

**Hypothesis** **6:** *The transparency of face masks will improve the ACC of micro-expression recognition but may prolong the RT for recognizing micro-expressions. Since transparent masks create partial interference with facial recognition and obscure parts of the face, they may lead to an increased RT for micro-expression recognition.*

**Hypothesis** **7:** *There is an interaction effect between background expressions and micro-expressions, as well as between mask types and micro-expressions. Under fear backgrounds, wearing a transparent mask may have a particularly negative impact on micro-expression recognition, potentially reducing the ACC and prolonging the RT. Fear backgrounds increase the emotional burden on individuals, exacerbating the difficulty of recognition due to facial expression obstruction.*

**Hypothesis** **8:** *Transparent face masks will enhance the favorability of micro-expression faces. Wearing opaque masks, on the other hand, will reduce the favorability of recognizing happy and neutral micro-expressions because positive facial expressions become difficult to discern, while it will increase the favorability of recognizing fear micro-expressions. This is because negative emotional faces are obscured, reducing feelings of aversion.*

### 3.1. Participants

Based on calculations from G*Power 3.1, with an effect size f = 0.25 and α = 0.05, a total sample size of 14 was needed. To ensure a sufficiently large sample size, this experiment ultimately recruited 40 participants, all of whom were college students (equal numbers of males and females, with an average age of 21.55 ± 2.59 years (M ± SD)). All participants were right-handed, with normal or corrected-to-normal vision, and had no history of psychiatric or neurological disorders. All participants signed an informed consent form prior to the experiment, acknowledging the purpose and procedures of the experiment, and ensuring the anonymity and confidentiality of the data. During the experimental process, if the participants felt uncomfortable, they could terminate the experiment and withdraw at any time, and the researchers would provide appropriate psychological support and counseling. All experimental data were processed anonymously and used solely for this study, without disclosure of the participants’ personal information. This study was conducted according to the ethical principles of the Declaration of Helsinki and received approval from the Research Ethics Committee of the School of Education Science of Yangzhou University (JKY-2023061301).

### 3.2. Materials

The experimental materials were derived from the NimStim Face Stimulus Set (Tottenham et al., 2009). The NimStim Face Stimulus Set is a standardized library of facial expression images used for the study of human emotional expression. Tottenham and colleagues published both background and micro-expression images within the NimStim Face Stimulus Set.

Background expressions included three types of expressions (happy, neutral, fear), with 18 images for each, totaling 54 images. Each set of facial expression images consisted of an equal number of male and female faces, with 8 male and 10 female images for each expression. Micro-expression images also included three types of expressions (happy, neutral, fear), with 18 images for each, totaling 54 images. Each set of facial expression images consisted of an equal number of male and female faces, with 8 male and 10 female images for each expression.

All facial images were available in two versions, namely wearing an opaque white mask and wearing a transparent mask, resulting in a total of 54 background expression images and 54 micro-expression images for use in the experiment. The experiment utilized E-prime 2.0 software for the presentation and recording of data.

### 3.3. Procedure

The experimental procedure consisted of two stages: a practice phase and a formal experimental phase. During the practice phase, the participants were provided with 18 practice trials to ensure that they fully understood the experimental procedures. Feedback was given for each trial in the training phase, whereas no feedback was provided during the formal experiment. The flowchart for the training phase was identical to that of the formal phase. Based on previous research, the formal phase comprised 6 blocks, with each block containing 60 trials, totaling 360 trials. The participants were allowed a 3 min break between every two blocks (Zhu et al., 2017). Throughout the experiment, the participants were first seated in front of a computer, adjusting their viewing distance to ensure that the distance between the participant and the screen was approximately 50 cm, guaranteeing visual comfort and the ACC of the experimental data.

**Figure 2:**
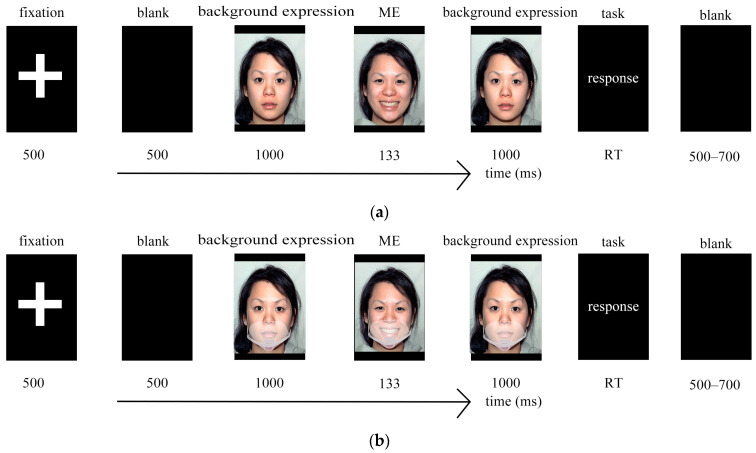
Flowchart of a single trial in Experiment 2 (**a**) illustrates the micro-expression recognition process without wearing a mask, while (**b**) shows the micro-expression recognition process with a transparent mask on).

The experimental procedure for each trial was as follows: First, a 500 ms fixation point “+” was presented in the center of the screen to focus the participants’ attention. Subsequently, a background expression (happy, neutral, fear) was displayed for 1000 ms. Next, a micro-expression face image (happy, neutral, fear) was rapidly presented for 133 ms. Then, the same background expression reappeared for 1000 ms. The participants were then required to discern the micro-expression that was rapidly presented between the preceding and following background expressions and select the type of micro-expression they saw on the screen. For selection, keyboard buttons were used: “A” indicated happy, “F” indicated neutral, and “J” indicated fear. After each micro-expression recognition task, a judgment of facial favorability followed immediately. The participants were required to rate the favorability of the face on a 9-point scale ranging from 1 to 9, with higher numbers indicating greater favorability. The numbers 1 through 9 on the keyboard corresponded to the scale points 1 through 9, respectively. After the button selection, a blank screen of 500 to 700 ms was presented before proceeding to the next trial (Figure 2).

### 3.4. Data Analysis

Data analysis was conducted using SPSS 25.0 software.

The experimental data included the ACC and RT of micro-expression recognition, and facial favorability ratings. A 3 (background expressions: happy, neutral, fear) × 3 (micro-expressions: happy, neutral, fear) × 2 (mask type: opaque white mask, transparent mask) repeated measures analysis of variance (ANOVA) was conducted to examine the effects of background expressions, types of micro-expressions, and mask types on the ACC and RT of micro-expression recognition, and facial favorability ratings. Results that did not meet the assumption of sphericity were corrected using the Greenhouse–Geisser correction method. All post hoc tests employed the Bonferroni correction. Partial eta squared ($\eta_{p}^{2}$) was used to describe the effect size. Independent variables included types of background expressions (happy, neutral, fear), types of micro-expressions (happy, neutral, fear), and mask types (wearing a mask, not wearing a mask); dependent variables were the ACC and RT of micro-expression recognition, and facial favorability ratings. RTs were recorded from the appearance of the judgment interface to the moment the participant pressed the corresponding key.

### 3.5. Results and Discussion

#### 3.5.1. The ACC of the Micro-Expression Recognition Task

The main effect of background expressions was not significant, with F(2,78) = 0.329, *p* = 0.721, and $\eta_{p}^{2}$ = 0.008; the main effect of micro-expressions was not significant, with F(2,78) = 0.023, *p* = 0.963, and $\eta_{p}^{2}$ = 0.001; the main effect of mask type was not significant, with F(1,39) = 0.423, *p* = 0.519, and $\eta_{p}^{2}$ = 0.011; the interaction effect between background expressions and micro-expressions was not significant, with F(4,156) = 1.634, *p* = 0.196, and $\eta_{p}^{2}$ = 0.040; the interaction effect between background expressions and mask type was not significant, with F(2,78) = 2.581, *p* = 0.092, and $\eta_{p}^{2}$ = 0.062; the interaction effect between micro-expressions and mask type was not significant, with F(2,78) = 3.382, *p* = 0.056, and $\eta_{p}^{2}$ = 0.080; and the three-way interaction effect among background expressions, micro-expressions, and mask type was not significant, with F(4,156) = 0.968, *p* = 0.427, and $\eta_{p}^{2}$ = 0.024.

Based on these data analysis results, it can be concluded that there were no significant differences in the combined effects of background expressions, micro-expressions, and mask transparency on the ACC of micro-expression recognition.

#### 3.5.2. The RT in the Micro-Expression Recognition Task

The main effect of background expressions was significant, with F(2,78) = 4.185, *p* = 0.019, and $\eta_{p}^{2}$ = 0.097. The post hoc test results indicated that compared with neutral background expressions (819.25 ± 362.31), the participants had a longer RT under fear background expressions (904.45 ± 431.12) (*p* = 0.018). Differences under other conditions were not significant (*p* > 0.05). This suggests that fear background expressions prolonged the duration for recognizing micro-expressions.

The main effect of micro-expressions was significant, with F(2,78) = 13.237, *p* < 0.001, and $\eta_{p}^{2}$ = 0.253. The post hoc test results indicated that compared with happy micro-expressions (792.63 ± 401.20), the participants had a longer RT under neutral micro-expressions (925.81 ± 470.93) (*p* < 0.001). Compared with fear micro-expressions (840.60 ± 363.65), the participants had a longer RT under neutral micro-expressions (925.81 ± 470.93) (*p* = 0.004). Differences under other conditions were not significant (*p* > 0.05). This indicates that fear and neutral micro-expressions prolonged the participants’ RTs for recognizing micro-expressions.

The main effect of mask type was significant, with F(1,39) = 14.302, *p* = 0.001, and $\eta_{p}^{2}$ = 0.268. The post hoc test results indicated that compared with wearing opaque masks (869.81 ± 435.36), the participants had a shorter RT when wearing transparent masks (809.21 ± 388.50) (*p* = 0.001). This suggests that wearing transparent masks helped to reduce the duration for recognizing micro-expressions, as the lower obstruction degree effectively assisted the participants in quickly recognizing micro-expressions.

The interaction effect between background expressions and micro-expressions was significant, with F(4,156) = 6.245, *p* < 0.001, and $\eta_{p}^{2}$ = 0.138. The interaction effect between micro-expressions and mask type was significant, with F(2,78) = 3.588, *p* = 0.069, and $\eta_{p}^{2}$ = 0.061.

Due to the significant interaction between background expressions and micro-expressions, simple effect analyses were conducted. The results are discussed below.

As can be seen from Table 5, the results of the first four groups were significant. These results indicate that individuals’ RTs for recognizing micro-expressions were influenced by the interaction between background expressions and micro-expressions. When wearing opaque masks, recognizing fear micro-expressions under fear background expressions resulted in a longer RT compared with happy and neutral background expressions. When wearing transparent masks, recognizing neutral micro-expressions under neutral background expressions resulted in a longer RT compared with happy background expressions; recognizing fear micro-expressions under fear background expressions resulted in a longer RT compared with neutral background expressions. This demonstrates the interaction effect of background expressions and micro-expressions, especially fear background expressions, which significantly prolonged individuals’ RT for recognizing micro-expressions, and the duration for recognizing different micro-expressions varied with changes in background expressions.

In summary, individuals’ RTs for recognizing micro-expressions, regardless of the type of mask worn, were significantly affected by background expressions and micro-expressions (MEs), as well as the interaction between these two factors.

Due to the significant interaction between mask type and micro-expressions, simple effect analyses were conducted. The results are discussed below.

As can be seen from Table 6, the results of the first four groups were significant. These results indicate that individuals’ RTs for recognizing micro-expressions were influenced by the interaction between mask type and micro-expressions. Against a happy background expression, recognizing neutral micro-expressions took longer with an opaque mask than with a transparent mask; against neutral and fear background expressions, recognizing happy micro-expressions took longer with an opaque mask than with a transparent mask; and against a fear background expression, recognizing fear micro-expressions took longer with an opaque mask than with a transparent mask. This demonstrates the interaction effect of micro-expressions and mask type, with RTs for recognizing micro-expressions varying according to mask type.

In summary, individuals’ RTs for recognizing micro-expressions under different background expressions were significantly affected by mask type and micro-expressions (MEs), as well as the interaction between these two factors.

The interaction effect between background expressions and mask type was not significant, with F(2,78) = 1.184, *p* = 0.304, and $\eta_{p}^{2}$ = 0.029. The main effect of background expressions, micro-expressions, and mask type was not significant, with F(4,156) = 2.540, *p* = 0.069, and $\eta_{p}^{2}$ = 0.061.

#### 3.5.3. The Favorability of Micro-Expression Face Recognition

The main effect of background expressions was significant, with F(2,78) = 11.680, *p* = 0.001, and $\eta_{p}^{2}$ = 0.230. The post hoc test results indicated that compared with fear background expressions (4.82 ± 1.09), the participants had higher favorability ratings for micro-expression recognition under happy background expressions (5.12 ± 1.07) (*p* = 0.003). Compared with fear background expressions (4.82 ± 1.09), the participants had higher favorability ratings for micro-expression recognition under neutral background expressions (5.02 ± 1.01) (*p* < 0.001). Differences under other conditions were not significant (*p* > 0.05). This suggests that fear background expressions reduced the favorability of micro-expression recognition. The main effect of micro-expressions was significant, with F(2,78) = 46.150, *p* < 0.001, and $\eta_{p}^{2}$ = 0.542. The post hoc test results indicated that compared with neutral micro-expressions (4.93 ± 0.85), the participants had higher favorability ratings for micro-expression recognition under happy micro-expressions (5.92 ± 1.17) (*p* < 0.001). Compared with fear micro-expressions (4.11 ± 1.15), the participants had higher favorability ratings for micro-expression recognition under happy micro-expressions (5.92 ± 1.17) (*p* < 0.001). Compared with fear micro-expressions (4.82 ± 1.09), the participants had higher favorability ratings for micro-expression recognition under neutral micro-expressions (4.93 ± 0.85) (*p* < 0.001). This indicates that fear micro-expressions also reduced the favorability of micro-expression recognition. The main effect of mask type was significant, with F(1,39) = 6.384, *p* = 0.016, and $\eta_{p}^{2}$ = 0.141. The post hoc test results indicated that compared with wearing opaque white masks (4.94 ± 1.03), the participants had higher favorability ratings for micro-expression recognition when wearing transparent masks (5.03 ± 1.08) (*p* = 0.016). This suggests that transparent masks can significantly enhance the favorability of facial micro-expression recognition.

The interaction effect between background expressions and micro-expressions was significant, with F(4,156) = 13.166, *p* < 0.001, and $\eta_{p}^{2}$ = 0.252. The interaction effect between micro-expressions and mask type was significant, with F(2,78) = 29.690, *p* < 0.001, and $\eta_{p}^{2}$ = 0.432. The interaction effect between background expressions and mask type was significant, with F(2,78) = 19.690, *p* = 0.013, and $\eta_{p}^{2}$ = 0.105.

Due to the significant interaction between background expressions and micro-expressions, simple effect analyses were conducted. The results are discussed below.

As can be seen from Table 7, the results of the first eleven groups were significant. These results indicate that individuals’ favorability ratings for recognizing micro-expressions were influenced by the interaction between background expressions and micro-expressions. When wearing an opaque mask, recognizing happy micro-expressions under a happy background expression resulted in higher favorability ratings than under neutral or fear background expressions; recognizing neutral micro-expressions under a happy background expression also resulted in higher favorability ratings than under neutral background expressions. When wearing a transparent mask, recognizing happy micro-expressions under both happy and neutral background expressions resulted in higher favorability ratings than under fear background expressions; recognizing neutral micro-expressions under a happy background expression resulted in higher favorability ratings than under neutral or fear background expressions; and recognizing fear micro-expressions under a neutral background expression resulted in higher favorability ratings than under fear background expressions. This demonstrates the impact of different background expressions on the recognition of micro-expressions, with favorability ratings generally being higher under happy background expressions than under neutral or fear background expressions.

In summary, individuals’ favorability ratings for recognizing micro-expressions, whether wearing opaque masks or transparent masks, were significantly affected by background expressions and micro-expressions (MEs), as well as the interaction between these two factors.

Due to the significant interaction between mask type and micro-expressions, simple effect analyses were conducted. The results are discussed below.

As can be seen from Table 8, the results of the first four groups were significant. These results indicate that when recognizing happy micro-expressions under happy, neutral, and fear background expressions, the favorability rating with a transparent mask was consistently higher than with an opaque mask, suggesting that transparent masks can significantly enhance the favorability of recognizing happy-valenced micro-expressions; meanwhile, when recognizing fear micro-expressions against a fear background expression, the favorability rating with an opaque white mask was higher than with a transparent mask, indicating that opaque masks can partially obscure facial expressions, thereby reducing the negative impact of recognizing fear micro-expressions. This reflects the interaction effect of micro-expressions and mask type, with the favorability of recognizing different micro-expressions varying according to mask type.

In summary, individuals’ favorability ratings for recognizing micro-expressions under different background expressions were significantly affected by mask type and micro-expressions (MEs), as well as the interaction between these two factors.

The main effect of background expressions, micro-expressions, and mask types was not significant, with F(4,156) =1.252, *p* = 0.292, and $\eta_{p}^{2}$ = 0.031. The interaction effect between micro-expressions and mask types was not significant, with F(2,78) = 2.966, *p* = 0.102, and $\eta_{p}^{2}$ = 0.023.

The analysis results from Experiment 2 indicate that the factor of background expressions significantly influenced the RT in micro-expression recognition but did not significantly affect ACC, which differs from Hypothesis 5. This may suggest that background expressions do not directly affect the ACC of micro-expression recognition but can increase the recognizability of micro-expressions, thereby prolonging the RT for recognition. The type of mask affected the RT in micro-expression recognition, with shorter RTs when wearing transparent masks, which is consistent with Hypothesis 6. In the RT for recognizing micro-expressions, the interaction effects between background expressions and micro-expressions, as well as between micro-expressions and mask types, were significant, which aligns with Hypothesis 7. When evaluating the favorability of micro-expressions, the main effects of background expressions, micro-expressions, and mask types were all significant, with fear background expressions reducing favorability, fear micro-expressions also reducing favorability, and wearing transparent masks increasing favorability, which is consistent with Hypothesis 8. The focus of Experiment 2 was to explore the impact of wearing masks with different degrees of facial obstruction on micro-expression recognition. Therefore, would wearing masks with patterns interfere with the recognition of micro-expressions? Based on this hypothesis, Experiment 3 of this study designed two types of masks, namely a plain white mask without patterns and a patterned mask (the pattern being neutral, i.e., patterns that do not elicit significant emotions), to reveal the hypotheses of Experiment 3 by comparing the wearing of these two types of masks. Experiment 3 also examined the ACC, RT, and favorability of micro-expression recognition.

## 4. Experiment 3: The Impact of Mask Surface Patterns on Micro-Expression Recognition

Experiment 3 employed a within-subjects design of 3 (background expressions: happy, neutral, fear) × 3 (micro-expressions: happy, neutral, fear) × 2 (mask types: plain white mask, patterned mask) to investigate the impact of different types of background expressions and mask wearing on micro-expression recognition and facial favorability. This experiment proposes the following hypotheses:

**Hypothesis** **9:** *The emotional valence of background expressions will affect the ACC of micro-expression recognition. Specifically, the ACC of micro-expression recognition will be highest under happy backgrounds and lowest under fear backgrounds. This is because positive background emotions can enhance an individual’s emotional recognition ability, while negative background emotions may increase the difficulty of recognition.*

**Hypothesis** **10:** *Patterned masks will reduce the ACC of micro-expression recognition and may prolong the RT for recognizing micro-expressions. Patterned masks create partial interference with facial recognition, which may lead to an increased RT and decreased ACC in micro-expression recognition.*

**Hypothesis** **11:** *There is an interaction effect between background expressions and micro-expressions, as well as between mask types and micro-expressions. Under fear backgrounds, wearing a plain white mask without patterns will have a particularly negative impact on micro-expression recognition, potentially reducing the ACC and prolonging the RT. Fear backgrounds increase the emotional burden on individuals, exacerbating the difficulty of recognition due to facial expression obstruction.*

**Hypothesis** **12:** *Patterned masks will enhance the favorability of micro-expression faces, especially for fear micro-expressions, as the presence of patterns may distract participants to some extent, thus affecting the favorability of micro-expression recognition. Wearing a plain white mask without patterns will reduce the favorability of recognizing happy and neutral micro-expressions, as positive facial expressions become more difficult to discern.*

The experiment was divided into two tasks: Task 1 was to judge the type of micro-expression, and Task 2 was to rate the favorability of micro-expressions.

The experimental procedure included two stages: a practice phase and a formal experimental phase. During the practice phase, the participants were provided with 18 practice trials to ensure they fully understood the experimental procedures. Feedback was given for each trial in the training phase, whereas no feedback was provided during the formal experiment. The flowchart for the training phase was identical to that of the formal phase. Based on previous research, the formal phase included 6 blocks, with each block containing 60 trials, totaling 360 trials. The participants were allowed a 3 min break between every two blocks.

Throughout the experiment, the participants were first seated in front of a computer, adjusting their viewing distance to ensure that the distance between the participant and the screen was approximately 50 cm to guarantee visual comfort and the ACC of the experimental data.

### 4.1. Participants

Based on calculations from G*Power 3.1, with an effect size f = 0.25 and α = 0.05, a total sample size of 14 was needed. To ensure a sufficiently large sample size, this experiment ultimately recruited 40 participants, all of whom were college students (equal numbers of males and females, with an average age of 21.3 ± 1.94 years (M ± SD)). All participants were right-handed, with normal or corrected-to-normal vision, and had no history of psychiatric or neurological disorders. All participants signed an informed consent form before the experiment, acknowledging the purpose and procedures of the experiment, and ensuring the anonymity and confidentiality of the data. During the experimental process, if the participants felt uncomfortable, they could terminate the experiment and withdraw at any time, and the researchers would provide appropriate psychological support and counseling. All experimental data were processed anonymously and used solely for this study, without disclosure of the participants’ personal information. This study was conducted according to the ethical principles of the Declaration of Helsinki and received approval from the Research Ethics Committee of the School of Education Science of Yangzhou University (JKY-2023061301).

### 4.2. Materials

The experimental materials were derived from the NimStim Face Stimulus Set (Tottenham et al., 2009). The NimStim Face Stimulus Set is a standardized library of facial expression images used for the study of human emotional expression. Tottenham and colleagues published both background and micro-expression images within the NimStim Face Stimulus Set.

Background expressions included three types of expressions (happy, neutral, fear), with 18 images for each, totaling 54 images. Each set of facial expression images consisted of an equal number of male and female faces, with 8 male and 10 female images for each expression. Micro-expression images also included three types of expressions (happy, neutral, fear), with 18 images for each, totaling 54 images. Each set of facial expression images consisted of an equal number of male and female faces, with 8 male and 10 female images for each expression.

All facial images were presented in two types of masks, namely white without patterns and with neutral patterns (the patterns on the patterned masks were neutral, i.e., not designs that would elicit strong emotional responses from the participants), resulting in a total of 54 background expression images and 54 micro-expression images for use in the experiment. The experiment utilized E-prime 2.0 software for the presentation and recording of data.

### 4.3. Procedure

The experimental procedure consisted of two stages: a practice phase and a formal experimental phase. During the practice phase, participants were provided with 18 practice trials to ensure that they fully understood the experimental procedures. Feedback was given for each trial in the training phase, whereas no feedback was provided during the formal experiment. The flowchart for the training phase was identical to that of the formal phase. Based on previous research (Zhu et al., 2017), the formal phase comprised 6 blocks, with each block containing 60 trials, totaling 360 trials. The participants were allowed a 3 min break between every two blocks. Throughout the experiment, the participants were first seated in front of a computer, adjusting their viewing distance to ensure that the distance between the participant and the screen was approximately 50 cm, guaranteeing visual comfort and the ACC of the experimental data.

**Figure 3:**
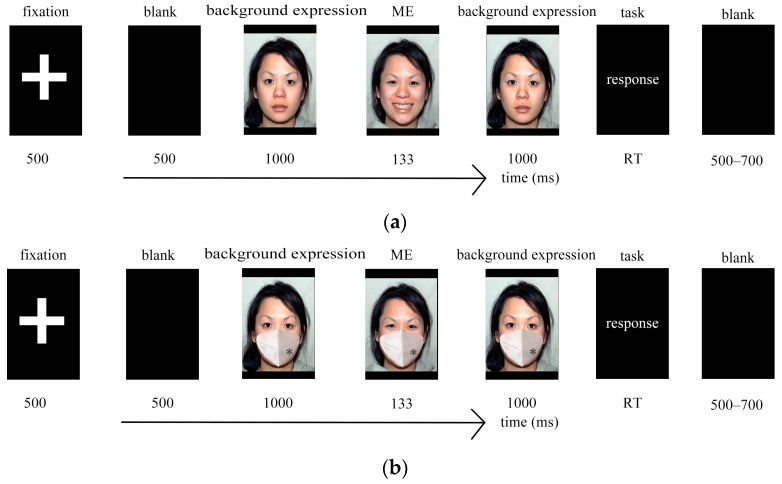
Flowchart of a single trial in Experiment 3 ((**a**) illustrates the micro-expression recognition process without wearing a mask, while (**b**) shows the micro-expression recognition process with a patterned mask on). The asterisk in the lower right corner of the mask surface indicates that this is a mask with a pattern.

The experimental procedure for each trial was as follows: First, a 500 ms fixation point “+” was presented in the center of the screen to focus the participants’ attention. Subsequently, a background expression (happy, neutral, fear) was displayed for 1000 ms. Next, a micro-expression face image (happy, neutral, fear) was rapidly presented for 133 ms. Then, the same background expression reappeared for 1000 ms. The participants were then required to discern the micro-expression that was rapidly presented between the preceding and following background expressions and select the type of micro-expression they saw on the screen. For selection, keyboard buttons were used: “A” indicated happy, “F” indicated neutral, and “J” indicated fear. After each micro-expression recognition task, a judgment of facial favorability followed immediately. The participants were required to rate the favorability of the face on a 9-point scale ranging from 1 to 9, with higher numbers indicating greater favorability. The numbers 1 through 9 on the keyboard corresponded to the scale points 1 through 9, respectively. After the button selection, a blank screen of 500 to 700 ms was presented before proceeding to the next trial (Figure 3).

### 4.4. Data Analysis

Data analysis was conducted using SPSS 25.0 software.

The experimental data included the ACC and RT of micro-expression recognition, and facial favorability ratings. A 3 (background expressions: happy, neutral, fear) × 3 (micro-expressions: happy, neutral, fear) × 2 (mask types: white mask, patterned mask) repeated measures analysis of variance (ANOVA) was conducted to examine the effects of background expressions, types of micro-expressions, and mask types on the ACC and RT of micro-expression recognition, and facial favorability ratings. Results that did not meet the assumption of sphericity were corrected using the Greenhouse–Geisser correction method. All post hoc tests employed the Bonferroni correction. Partial eta squared ($\eta_{p}^{2}$) was used to describe the effect size. Independent variables included types of background expressions (happy, neutral, fear), types of micro-expressions (happy, neutral, fear), and mask types (wearing a white mask, wearing a patterned mask); dependent variables were the ACC and RT of micro-expression recognition, and facial favorability ratings. RTs were recorded from the appearance of the judgment interface to the moment the participant pressed the corresponding key.

### 4.5. Results and Discussion

#### 4.5.1. The ACC of the Micro-Expression Recognition Task

The main effect of background expressions was significant, with F(2,78) = 29.769, *p* < 0.001, and $\eta_{p}^{2}$ = 0.433. The post hoc test results indicated that compared with a happy background expression (0.76 ± 0.19), the participants had higher ACC rates under neutral background expressions (0.84 ± 0.17) (*p* < 0.001). Compared with a fear background expression (0.74 ± 0.21), the participants had higher ACC rates under neutral background expressions (0.84 ± 0.17) (*p* < 0.001). Differences under other conditions were not significant (*p* > 0.05).

The interaction effect between background expressions and micro-expressions was significant, with F(4,156) = 27.844, *p* < 0.001, and $\eta_{p}^{2}$ = 0.417; the interaction effect between micro-expressions and mask types was significant, with F(2,78) = 4.711, *p* = 0.012, and $\eta_{p}^{2}$ = 0.108.

Due to the significant interaction between background expressions and micro-expressions, simple effect analyses were conducted. The results are discussed below.

As can be seen from Table 9, the results of the first nine groups were significant. These results indicate that individuals’ ACC rates in recognizing micro-expressions were influenced by the interaction between background expressions and micro-expressions. When wearing a mask without a pattern, the ACC rate for recognizing happy micro-expressions was higher under happy and neutral background expressions than under fear background expressions; for recognizing neutral micro-expressions, the ACC rate was higher under neutral background expressions than under happy background expressions. When wearing a patterned mask, the ACC rate for recognizing happy micro-expressions was higher under happy background expressions than under neutral or fear background expressions; for recognizing neutral micro-expressions, the ACC rate was higher under neutral background expressions than under happy or fear background expressions, yet the ACC rate under fear background expressions was higher than under happy background expressions. It is evident that the ACC rate for recognizing micro-expressions was influenced by the interaction between background expressions and micro-expressions.

Due to the significant interaction between mask type and micro-expressions, simple effect analyses were applied. The results are discussed below.

As can be seen from Table 10, the results of the first group were significant. This suggests that the ACC rate of recognizing micro-expressions varied with changes in mask type and micro-expressions, especially when wearing a patterned mask, which enhanced the ACC rate for recognizing happy micro-expressions.

The main effect of micro-expressions was not significant, with F(2,78) = 2.254, *p* = 0.121, and $\eta_{p}^{2}$ = 0.055; the main effect of mask type was not significant, with F(1,39) = 0.120, *p* = 0.731, and $\eta_{p}^{2}$ = 0.003; the interaction effect between background expressions and mask type was not significant, with F(2,78) = 0.526, *p* = 0.593, and $\eta_{p}^{2}$ = 0.013; and the three-way interaction effect among background expressions, micro-expressions, and mask type was not significant, with F(4,156) = 0.759, *p* = 0.526, and $\eta_{p}^{2}$ = 0.019.

#### 4.5.2. The RT in the Micro-Expression Recognition Task

The main effect of background expressions was significant, with F(2,78) = 4.232, *p* = 0.018, and $\eta_{p}^{2}$ = 0.098.

The post hoc test results indicated that compared with neutral background expressions (863.94 ± 408.14), the participants had a longer RT under fear background expressions (929.80 ± 441.78) (*p* = 0.031). Differences under other conditions were not significant (*p* > 0.05). This suggests that background expressions influence the RT for recognizing micro-expressions, with fear backgrounds increasing the duration required for recognition.

The main effect of micro-expressions was significant, with F(2,78) = 7.693, *p* = 0.001, and $\eta_{p}^{2}$ = 0.165.

The post hoc test results indicated that compared with neutral micro-expressions (948.91 ± 458.10), the participants had a shorter RT under happy micro-expressions (861.67 ± 407.85) (*p* = 0.004). Compared with neutral micro-expressions (948.91 ± 458.10), the participants also had a shorter RT under fear micro-expressions (861.74 ± 437.11) (*p* = 0.004). Differences under other conditions were not significant (*p* > 0.05). This indicates that micro-expressions influenced the RT for micro-expression recognition.

The interaction effect between background expressions and micro-expressions was significant, with F(4,156) = 7.786, *p* < 0.001, and $\eta_{p}^{2}$ = 0.166.

Due to the significant interaction between background expressions and micro-expressions, simple effect analyses were conducted. The results are discussed below.

As can be seen from Table 11, the results of the first four groups were significant. These results indicate that individuals’ RTs for recognizing micro-expressions were influenced by the interaction between background expressions and micro-expressions. When wearing a plain white mask, the RT for recognizing fear micro-expressions under fear background expressions was longer than under happy background expressions and also longer than under neutral background expressions. When wearing a patterned mask, the RT for recognizing fear micro-expressions under fear background expressions was similarly longer than under happy background expressions and neutral background expressions.

This suggests that individuals’ RTs for recognizing micro-expressions when wearing different types of masks were significantly influenced by background expressions and micro-expressions, as well as the interaction between these two factors.

The main effect of mask type was not significant, with F(1,39) = 0.000, *p* = 0.997, and $\eta_{p}^{2}$ < 0.001. The interaction effect between background expressions and mask type was not significant, with F(2,78) = 1.855, *p* = 0.163, and $\eta_{p}^{2}$ = 0.045. The interaction effect between micro-expressions and mask type was not significant, with F(2,78) = 0.469, *p* = 0.627, and $\eta_{p}^{2}$ = 0.012. The three-way interaction effect among background expressions, micro-expressions, and mask type was not significant, with F(2,156) = 0.608, *p* = 0.632, and $\eta_{p}^{2}$ = 0.015.

#### 4.5.3. The Favorability of Micro-Expression Face Recognition

The main effect of background expressions was significant, with F(2,78) = 7.915, *p* = 0.002, and $\eta_{p}^{2}$ = 0.169. The post hoc test results indicated that compared with fear background expressions (4.86 ± 1.20), the participants had higher favorability ratings under happy background expressions (5.11 ± 1.13) (*p* = 0.010). Compared with fear background expressions (4.86 ± 1.20), the participants had higher favorability ratings under neutral background expressions (5.03 ± 1.11) (*p* = 0.010). Differences under other conditions were not significant (*p* > 0.05).

The main effect of micro-expressions was significant, with F(2,78) = 42.145, *p* < 0.001, and $\eta_{p}^{2}$ = 0.519. The post hoc test results indicated that compared with neutral micro-expressions (4.49 ± 1.08), the participants had higher favorability ratings under happy micro-expressions (5.86 ± 1.16) (*p* < 0.001). Compared with fear micro-expressions (4.19±1.22), the participants had higher favorability ratings under happy micro-expressions (5.86 ± 1.16) (*p* < 0.001). Compared with fear micro-expressions (4.19 ± 1.22), the participants had higher favorability ratings under neutral micro-expressions (4.49 ± 1.08) (*p* < 0.001).

The main effect of mask type was significant, with F(1,39) = 4.364, *p* = 0.043, and $\eta_{p}^{2}$ = 0.101. The post hoc test results indicated that compared with patterned masks (4.98 ± 1.15), the participants had higher favorability ratings under plain masks without patterns (5.01 ± 1.15) (*p* < 0.001).

The interaction effect between background expressions and micro-expressions was significant, with F(4,156) = 11.150, *p* < 0.001, and $\eta_{p}^{2}$ = 0.222; the three-way interaction effect among background expressions, micro-expressions, and mask type was significant, with F(4,156) = 2.917, *p* = 0.023, and $\eta_{p}^{2}$ = 0.070.

Due to the significant interaction between background expressions and micro-expressions, simple effect analyses were conducted. The results are discussed below.

As can be seen from Table 12, the results of the first six groups were significant. These results indicate that individuals’ favorability ratings for recognizing micro-expressions were influenced by the interaction between background expressions and micro-expressions. When wearing masks with and without patterns, the favorability rating for recognizing happy micro-expressions was higher under happy background expressions than under neutral background expressions, and higher under neutral background expressions than under fear background expressions. Therefore, the background had a particularly significant impact on the recognition of happy micro-expressions.

Due to the significant interaction effect among background expressions, micro-expressions, and mask types, with F(4,156) = 2.917, *p* = 0.023, and $\eta_{p}^{2}$ = 0.070, simple effect analyses of background expressions and mask types were conducted. The results are discussed below.

As can be seen from Table 13, the results of the first two groups were significant. These results indicate that individuals’ favorability ratings for recognizing micro-expressions were influenced by the interaction between micro-expressions and mask types. 

The interaction effect between background expressions and masks was not significant, with F(2,78) = 0.491, *p* = 0.614, and $\eta_{p}^{2}$ = 0.012; the interaction effect between micro-expressions and masks was not significant, with F(2,78) = 0.229, *p* = 0.796, and $\eta_{p}^{2}$ = 0.006.

The analysis results from Experiment 3 indicate that the factor of background expressions significantly influenced the ACC of micro-expression recognition, which is consistent with Hypothesis 9 that the emotional valence of background expressions would affect the ACC of micro-expression recognition. However, the ACC was the highest under neutral background expressions, and fear and happy background expressions reduced the ACC of micro-expression recognition, which differs from Hypothesis 9’s prediction that fear background expressions would decrease the ACC. A possible explanation is that fear background expressions stimulate individuals’ attention to recognize micro-expressions, hence the higher ACC under fear expressions. The impact of background expressions on the RT for micro-expression recognition was also significant, with longer RTs under fear background expressions. Additionally, when recognizing happy micro-expressions against a neutral background, the ACC with patterned masks was significantly higher than with plain masks, while the presence of patterns on masks did not significantly affect the RT. This is consistent with Hypothesis 10 regarding the ACC but differs regarding the RT, possibly because mask patterns do not influence the overall RT for recognizing micro-expressions, and neutral patterns are less likely to evoke emotional changes in participants. These results confirmed the interaction effect among background expressions, micro-expressions, and mask types as posited in Hypothesis 11. Patterned masks also enhanced the favorability of micro-expression faces, which is in line with Hypothesis 12.

## 5. General Discussion

The results of Experiment 1 indicated that wearing face masks had a significant impact on the RT of micro-expression recognition, but not on the ACC. There was a significant interaction between background expressions and the state of mask wearing. These findings partially aligned with the hypotheses, showing discrepancies in ACC predictions but confirming the hypotheses related to the RT. Notably, the adverse impact on the RT was more pronounced when wearing masks over fear background expressions. Additionally, facial attractiveness ratings were the highest for happy background expressions and the lowest for fear ones, with mask wearing generally resulting in lower ratings. The results suggest that wearing masks, especially under fear background expressions, negatively affects micro-expression recognition and facial attractiveness, consistent with the corresponding hypotheses. Experiment 1 focused on the impact of mask wearing on micro-expression recognition, which led to further investigations into whether mask opacity would affect this recognition, thus prompting the design of Experiment 2.

Experiment 2 explored the impact of different mask opacities on micro-expression recognition, considering background expressions, micro-expressions, and facial attractiveness. The results showed that background expressions significantly affected the RT but not the ACC, indicating that they influence the discriminability of recognition rather than the direct ACC. Mask type affected the RT, with transparent masks leading to shorter times, consistent with the hypotheses. Significant interactions were found between background expressions and micro-expressions, as well as between micro-expressions and mask types, in terms of the RT. In terms of facial attractiveness, the main effects of background expressions, micro-expressions, and mask types were significant, with fear expressions and micro-expressions reducing attractiveness, while transparent masks increased it. The focus on mask opacity in Experiment 2 raised the question of whether patterned masks would interfere with micro-expression recognition, leading to the design of Experiment 3.

In Experiment 3, the effects of plain white masks and patterned (neutral) masks on micro-expression recognition were compared. The results showed that, in terms of accuracy, overall, the highest accuracy was observed under neutral background conditions. The accuracy under neutral background expressions was significantly higher than that under happy and fearful background expressions. A possible explanation is that the participants experienced aversion due to the negative emotional stimulation under fearful background expressions, which diverted their attention and made it difficult to accurately identify the type of micro-expression hidden within. In contrast, when recognizing happy micro-expressions under neutral background expressions, the accuracy with patterned masks was significantly higher than that with white masks. A possible explanation is that when recognizing happy micro-expressions, positive emotions may make participants more willing to attend to additional facial information, and the pattern helps focus their attention, thereby enabling more accurate recognition of the micro-expression type. In terms of the reaction time (RT), longer RTs were observed under fearful background expressions compared to neutral background expressions. This may be explained by a similar mechanism to that observed for accuracy: participants experiencing aversion due to negative emotional stimulation under fearful background expressions may have their attention diverted, requiring more time to identify the type of micro-expression hidden within.

Comparing these three experiments, while wearing masks consistently affected the RT in micro-expression recognition, its impact on the ACC varied. The influence of background expressions on recognition was stable across the experiments, with fear expressions often prolonging the RT. Introducing mask opacity and patterns provided nuanced differences, revealing that transparent masks helped to accelerate recognition speeds, and patterned masks enhanced facial attractiveness and, in some contexts, improved recognition ACC.

### 5.1. The Impact of Face Mask Wearing on Micro-Expression Recognition

The experimental results indicate that wearing face masks significantly affected the RT in micro-expression recognition, particularly against fear backgrounds. The wearing of masks significantly prolonged the RT, possibly because fear backgrounds increase the emotional burden on individuals, making the obstruction of facial expressions further exacerbate the difficulty of recognition. Additionally, wearing masks significantly reduced facial favorability ratings, especially under negative contexts. These results suggest that wearing masks may have a negative impact on social interactions, particularly in emotionally negative situations.

Previous research found that when observing the whole body, the ACC of facial expression recognition was not significantly affected by masks, except for happy expressions. Specifically, for expressions of anger, sadness, and fear, the recognition ACC did not significantly decrease even when the face was obscured by a mask. However, for the recognition of happy expressions, the ACC significantly decreased when the face was obscured by a mask (Carbon, 2020). Another study found that wearing masks reduced the ACC of facial expression recognition, particularly for the recognition of disgust expressions (Grahlow et al., 2022). Compared with existing studies, the current study more comprehensively examines the impact of different types of masks on micro-expression recognition, which can be used to compare previous studies on ordinary expression recognition to discover the similarities and differences between micro-expression recognition and ordinary expression recognition. For instance, in this study, wearing masks did not significantly affect the ACC of micro-expression recognition, which differs from some results showing that masks can reduce the ACC of recognizing ordinary expressions. Furthermore, Grundmann’s research found that masks reduced the ACC of emotional expression recognition and made people feel less close to others (Grundmann et al., 2021), while this study further reveals the particularly significant impact of masks on micro-expression recognition under fear contexts, emphasizing the importance of situational factors. The negative impact of mask wearing on facial favorability is more pronounced in negative contexts, which is rarely mentioned in the existing literature.

### 5.2. The Impact of Mask Transparency on Micro-Expression Recognition

The experimental results show that the transparency level of face masks has a significant impact on the RT for micro-expression recognition. Under fear backgrounds, opaque masks significantly prolonged the RT, indicating that opaque masks may obscure the subtle changes in micro-expressions, increasing the difficulty of recognition. Facial favorability ratings were significantly higher under transparent mask conditions than under opaque mask conditions, especially against neutral and fear backgrounds. This suggests that transparent masks may mitigate the obstructive effects of facial expressions to some extent, reducing their negative impact on social interactions.

This study also considered the favorability of facial micro-expressions and found that transparent masks significantly enhanced facial favorability under both neutral and fear backgrounds. Previous researchers have found that transparent masks can restore key parts of the face that convey emotional information, thereby reducing communication barriers. There is a significant difference between transparent and opaque masks in the recognition of facial emotions. Opaque masks significantly reduce the ability to recognize facial emotions, especially in recognizing basic emotions. For example, studies have found that when the face is obscured by an opaque mask, people are most impaired in recognizing disgust, while the recognition of fear is least affected.

In contrast, transparent masks allow visible parts of facial expressions, especially those key areas that convey emotional information, such as the eyes and mouth, to remain visible. Therefore, transparent masks can restore the ability to recognize basic emotions, making emotional recognition comparable to when no mask is worn. For instance, a study by Marini et al. found that emotional recognition under transparent masks was as effective as without masks (Marini et al., 2021).

Transparent masks have a clear advantage over opaque masks in facial emotion recognition, significantly improving emotional recognition and understanding, thereby contributing to improved communication quality (McCrackin & Ristic, 2024).

### 5.3. The Impact of Mask Patterns on Micro-Expression Recognition

The experimental results indicate that the presence or absence of patterns on the masks did not significantly affect the RT and ACC rates of micro-expression recognition. This may be because the patterns were neutral in color and did not elicit significant emotional responses from the individuals, thus not significantly impacting the recognition of micro-expressions. Facial favorability ratings under plain mask conditions were significantly higher than under patterned mask conditions, especially against neutral and fear backgrounds. This suggests that patterns on masks may further reduce individuals’ favorability of faces, particularly in emotionally negative contexts.

Compared with Freud et al., who only explored the impact of patterns on attention allocation(Freud et al., 2020), our study delved into the dual effects of patterns on micro-expression recognition and favorability, revealing the significant negative effect of patterns on favorability in negative contexts.

### 5.4. The Combined Impact of Background Expressions, Micro-Expressions, and Mask Attributes on Micro-Expression Recognition

In all three experiments, significant interactions were observed between background expressions and mask attributes (wearing or not, transparency, and presence or absence of patterns), indicating that the impact of masks on micro-expression recognition and facial favorability varies across different contexts. Particularly under fear backgrounds, the negative impact of masks is more pronounced. This suggests that in emotionally negative contexts, facial obstruction may exacerbate individuals’ negative experiences, further affecting the effectiveness of social interactions.

This study combined the Brief Affect Recognition Test (BART) paradigm and the improved Japanese and Caucasian Brief Affect Recognition Test (JACBART) paradigm to conduct a series of experiments examining the cognitive mechanisms by which clue information influences facial recognition, especially the impact of wearing masks and specific types of masks on micro-expression recognition, enhancing the ecological validity of micro-expression recognition research. This study employed experimental psychological methods to verify the effects and impacts of recognizing micro-expressions under facial obstruction, particularly the changes in facial favorability under different types of masks, designing innovative experiments to promote and improve interpersonal relationships and interpersonal harmony.

Micro-expression recognition has also garnered significant interest in the healthcare domain, particularly due to its potential to reveal concealed emotions and underlying psychological states. Recent studies have shown that micro-expressions can provide critical insights into the emotional states of patients with various mental health conditions. For instance, research by Frank and Ekman demonstrated that micro-expressions can reveal emotions that individuals may be consciously or unconsciously concealing, which is particularly useful in the diagnosis and management of depression and other mood disorders (Frank & Ekman, 1997). These fleeting facial expressions can serve as early indicators of emotional distress, allowing clinicians to provide timely interventions.

Moreover, micro-expression recognition has been used to improve empathy and emotion recognition skills in patients with schizophrenia. Studies such as those by Krumhuber et al. have shown that training individuals with schizophrenia to recognize micro-expressions can enhance their ability to perceive emotions in others, which is crucial for social interaction and communication (Krumhuber et al., 2013). This application not only aids in the rehabilitation of patients but also supports their integration into social environments.

In addition, micro-expression recognition can be a valuable tool in the assessment of neurological disorders such as Parkinson’s disease. Research by Hager et al. has shown that facial muscle movements and micro-expressions can provide early indicators of such conditions, allowing for earlier diagnosis and intervention (Hager et al., 2001). This application highlights the potential of micro-expression recognition in both mental and neurological healthcare settings.

In law enforcement, micro-expression recognition has emerged as a powerful tool for deception detection and suspect evaluation. The ability to detect concealed emotions through micro-expressions can significantly enhance the effectiveness of interrogations and investigations. This capability allows law enforcement officers to identify suspects who may be withholding information or lying about their involvement in criminal activities.

Moreover, micro-expression recognition can be used in patrol and surveillance settings to identify individuals who exhibit signs of nervousness or deception. Studies by Matsumoto and Hwang have demonstrated that officers can use micro-expression recognition to make more informed decisions during field operations (Matsumoto & Hwang, 2011). The application of micro-expression recognition in law enforcement not only improves the accuracy of suspect evaluations but also enhances the overall efficiency of criminal investigations.

In summary, micro-expression recognition offers practical applications in both healthcare and law enforcement domains. It provides valuable insights into concealed emotions and underlying psychological states, aiding in the diagnosis and management of mental health conditions and enhancing the effectiveness of law enforcement activities. Future research should continue to explore the potential of micro-expression recognition in these and other domains, while also addressing the challenges of the real-time detection and interpretation of these subtle facial expressions.

While this study contributes to expanding our understanding of the relationship between mask wearing and micro-expression recognition, it has some limitations. First, this study only examined background expressions and micro-expressions of three emotional face types, namely happy, neutral, and fear, without examining more types of emotional facial expressions (sadness, disgust, surprise, etc.). Future research could explore the impact of a broader range of emotional facial expressions on micro-expression recognition. Second, the facial materials used in this study predominantly featured Western faces and lacked Eastern faces (especially Chinese faces), and the ACC and favorability of recognizing facial expressions of different ethnicities can vary culturally. Therefore, future studies could use more localized facial materials. Third, this study only investigated the facial obstruction caused by masks, while the impact of other facial obstructions, such as glasses, sunglasses, hats, and hair near the face, remains to be studied.

## 6. Conclusions

(1) The wearing of face masks significantly affects the RT and ACC of micro-expression recognition: particularly against fear backgrounds, wearing masks significantly prolonged the RT and reduced recognition ACC.This conclusion is consistent with the results of previous studies that wearing masks results in a decrease in face recognition ability, and the processing of masked faces is more dependent on local features rather than the overall configuration(Freud et al., 2020).

(2) The transparency of face masks has a significant impact on the RT of micro-expression recognition: opaque masks increased the difficulty of recognition, while transparent masks mitigated the negative effects of facial obstruction to some extent.

(3) The impact of mask surface patterns on micro-expression recognition is minimal; however, in negative contexts, patterns added visual interference, significantly prolonging the RT and increasing favorability ratings.

(4) Background expressions play a significant moderating role in the process of micro-expression recognition: the RTs were the longest and the ACC was the lowest under fear backgrounds, with significant interactions observed between background expressions and mask attributes.

## Figures and Tables

**Table 1:** In the analysis of reaction time (RT) in Experiment 1, “a” represents the background expression, with “a1” indicating a happy background expression, “a2” indicating a neutral background expression, and “a3” indicating a fearful background expression; “b” represents the micro-expression, with “b1” indicating a happy micro-expression, “b2” indicating a neutral micro-expression, and “b3” indicating a fearful micro-expression; “c” represents the mask, with “c1” indicating wearing a mask and “c2” indicating not wearing a mask. For example, if the *p*-value under “a2b2c2-a1b2c2” is significant, it means that the reaction time for identifying neutral micro-expressions under neutral background expressions without wearing a mask is significantly longer than that for identifying neutral micro-expressions under happy background expressions without wearing a mask. “M1–M2” represents the difference in means between two conditions. “SE” stands for the standard error. *p* < 0.05 stands for significant.

Judgment Mode	M1–M2	SE	*p*
a2b2c2-a1b2c2	316.216	62.549	<0.001
a2b1c2-a3b2c2	286.939	77.530	0.001
a3b3c1-a1b3c1	292.106	74.418	<0.001
a3b3c1-a2b3c1	253.815	69.707	0.001
a3b3c2-a1b3c2	239.89	86.586	0.009
a3b3c2-a2b3c2	217.387	80.320	0.010
a1b1c1-a2b1c1	11.443	73.872	0.878
a1b1c1-a3b1c1	50.197	68.891	0.471
a2b1c1-a3b1c1	38.755	74.316	0.605
a1b1c2-a2b1c2	103.992	83.892	0.223
a1b1c2-a3b1c2	100.881	53.052	0.065
a3b1c2-a2b1c2	3.111	85.757	0.971
a1b2c1-a2b2c1	46.068	83.471	0.584
a1b2c1-a3b2c1	67.599	81.477	0.412
a2b2c1-a3b2c1	21.531	61.765	0.729
a3b1c2-a1b2c2	29.277	71.487	0.684
a2b3c1-a1b3c1	38.291	63.840	0.552
a2b3c2-a1b3c2	22.503	71.326	0.754

**Table 2:** In the analysis of reaction time (RT) in Experiment 1, “a” represents the background expression, with “a1” indicating a happy background expression, “a2” indicating a neutral background expression, and “a3” indicating a fearful background expression; “b” represents the micro-expression, with “b1” indicating a happy micro-expression, “b2” indicating a neutral micro-expression, and “b3” indicating a fearful micro-expression; “c” represents the mask, with “c1” indicating wearing a mask and “c2” indicating not wearing a mask. For example, if the *p*-value under “a1b1c1-a1b1c2” is significant, it means that the reaction time for identifying happy micro-expressions under happy background expressions with a mask is significantly longer than that for identifying happy micro-expressions under happy background expressions without a mask. “M1–M2” represents the difference in means between two conditions. “SE” stands for the standard error. *p* < 0.05 stands for significant.

Judgment Mode	M1–M2	SE	*p*
a1b1c1-a1b1c2	127.694	52.127	0.019
a1b2c1-a1b2c2	300.154	74.658	<0.001
a2b1c1-a2b1c2	220.243	91.009	0.020
a3b1c1-a3b1c2	178.377	68.386	0.013
a3b2c1-a3b2c2	203.278	71.916	0.007
a1b3c2-a1b3c1	3.859	64.267	0.952
a2b2c2-a2b2c1	62.130	53.634	0.254
a2b3c1-a2b3c2	11.930	85.646	0.890
a3b3c1-a3b3c2	48.358	86.741	0.580

**Table 3:** In the analysis of favorability in Experiment 1, “a” represents the background expression, with “a1” indicating a happy background expression, “a2” indicating a neutral background expression, and “a3” indicating a fearful background expression; “b” represents the micro-expression, with “b1” indicating a happy micro-expression, “b2” indicating a neutral micro-expression, and “b3” indicating a fearful micro-expression; “c” represents the mask, with “c1” indicating wearing a mask and “c2” indicating not wearing a mask. For example, if the *p*-value under “a1b1c1-a2b1c1” is significant, it means that the favorability of identifying happy micro-expressions under happy background expressions with a mask is significantly higher than that of identifying happy micro-expressions under neutral background expressions with a mask. “M1–M2” represents the difference in means between two conditions. “SE” stands for the standard error. *p* < 0.05 stands for significant.

Judgment Mode	M1–M2	SE	*p*
a1b1c1-a2b1c1	0.539	0.113	<0.001
a1b1c1-a3b1c1	0.920	0.145	<0.001
a2b1c1-a3b1c1	0.381	0.112	0.002
a1b1c2-a2b1c2	0.545	0.142	<0.001
a1b1c2-a3b1c2	0.879	0.178	<0.001
a2b1c2-a3b1c2	0.334	0.070	<0.001
a2b2c1-a3b2c1	0.205	0.071	0.006
a1b3c2-a3b3c2	0.423	0.131	0.003
a2b3c2-a3b3c2	0.284	0.076	0.001
a1b2c1-a3b2c1	0.108	0.119	0.374
a2b2c1-a1b2c1	0.097	0.097	0.322
a1b1c2-a3b2c2	0.141	0.152	0.358
a2b2c2-a1b2c2	0.001	0.096	0.990
a2b2c2-a3b2c2	0.143	0.093	0.134
a1b3c1-a2b3c1	0.095	0.078	0.233
a1b3c1-a3b3c1	0.265	0.133	0.054
a2b3c1-a3b3c1	0.170	0.095	0.081
a1b3c2-a2b3c2	0.139	0.087	0.120

**Table 4:** In the analysis of favorability in Experiment 1, “a” represents the background expression, with “a1” indicating a happy background expression, “a2” indicating a neutral background expression, and “a3” indicating a fearful background expression; “b” represents the micro-expression, with “b1” indicating a happy micro-expression, “b2” indicating a neutral micro-expression, and “b3” indicating a fearful micro-expression; “c” represents the mask, with “c1” indicating wearing a mask and “c2” indicating not wearing a mask. For example, if the *p*-value under “a1b1c2-a1b1c1” is significant, it means that the favorability of identifying happy micro-expressions under happy background expressions without a mask is significantly higher than that of identifying happy micro-expressions under happy background expressions with a mask. “M1–M2” represents the difference in means between two conditions. “SE” stands for the standard error. *p* < 0.05 stands for significant.

Judgment Mode	M1–M2	SE	*p*
a1b1c2-a1b1c1	0.448	0.112	<0.001
a2b1c2-a2b1c1	0.441	0.130	0.002
a3b1c2-a3b1c1	0.489	0.105	<0.001
a3b3c1-a3b3c2	0.239	0.095	0.016
a1b2c2-a1b2c1	0.038	0.075	0.622
a1b3c1-a1b3c2	0.081	0.100	0.419
a2b2c1-a2b2c2	0.059	0.062	0.352
a2b3c1-a2b3c2	0.125	0.087	0.160
a3b2c2-a3b2c1	0.004	0.068	0.957

**Table 5:** In the analysis of reaction time (RT) in Experiment 2, “a” represents the background expression, with “a1” indicating a happy background expression, “a2” indicating a neutral background expression, and “a3” indicating a fearful background expression; “b” represents the micro-expression, with “b1” indicating a happy micro-expression, “b2” indicating a neutral micro-expression, and “b3” indicating a fearful micro-expression; “c” represents the mask, with “c1” indicating wearing a white mask and “c2” indicating wearing a transparent mask. For example, if the *p*-value under “a2b2c2-a1b2c2” is significant, it means that the reaction time for identifying neutral micro-expressions under neutral background expressions with a transparent mask is significantly longer than that for identifying neutral micro-expressions under happy background expressions with a transparent mask. “M1–M2” represents the difference in means between two conditions. “SE” stands for the standard error. *p* < 0.05 stands for significant.

Judgment Mode	M1–M2	SE	*p*
a2b2c2-a1b2c2	97.142	46.610	0.044
a3b3c1-a1b3c1	330.546	42.005	<0.001
a3b3c1-a2b3c1	252.589	51.068	<0.001
a3b3c2-a1b3c2	223.095	46.122	<0.001
a1b1c1-a2b1c1	96.126	47.906	0.052
a1b1c1-a3b1c1	17.600	58.824	0.766
a3b1c1-a2b1c1	78.526	61.329	0.208
a1b1c2-a2b1c2	118.029	75.022	0.124
a1b1c2-a3b1c2	44.041	80.312	0.587
a3b1c2-a2b1c2	73.987	51.621	0.160
a1b2c1-a2b2c1	160.754	121.358	0.193
a1b2c1-a3b2c1	163.231	129.412	0.215
a2b2c1-a3b2c1	2.477	61.032	0.968
a2b2c2-a3b2c2	11.292	80.514	0.889
a3b2c2-a1b2c2	85.850	78.951	0.284
a2b3c1-a1b3c1	77.957	39.939	0.058
a2b3c2-a1b3c2	103.283	56.457	0.075
a3b3c2-a2b3c2	119.812	65.339	0.074

**Table 6:** In the analysis of reaction time (RT) in Experiment 2, “a” represents the background expression, with “a1” indicating a happy background expression, “a2” indicating a neutral background expression, and “a3” indicating a fearful background expression; “b” represents the micro-expression, with “b1” indicating a happy micro-expression, “b2” indicating a neutral micro-expression, and “b3” indicating a fearful micro-expression; “c” represents the mask, with “c1” indicating wearing a white opaque mask and “c2” indicating wearing a transparent mask. For example, if the *p*-value under “a1b2c1-a1b2c2” is significant, it means that the reaction time for identifying neutral micro-expressions under happy background expressions with a white opaque mask is significantly longer than that for identifying neutral micro-expressions under happy background expressions with a transparent mask. “M1–M2” represents the difference in means between two conditions. “SE” stands for the standard error. *p* < 0.05 stands for significant.

Judgment Mode	M1–M2	SD	*p*
a1b2c1-a1b2c2	309.051	126.311	0.019
a2b1c1-a2b1c2	110.470	37.945	0.006
a3b1c1-a3b1c2	115.009	55.058	0.043
a3b3c1-a3b3c2	98.130	39.008	0.016
a1b1c1-a1b1c2	88.568	65.885	0.187
a1b3c2-a1b3c1	9.321	41.182	0.822
a2b2c1-a2b2c2	51.155	51.316	0.325
a2b3c2-a2b3c1	34.646	53.683	0.522
a3b2c1-a3b2c2	59.970	63.420	0.350

**Table 7:** In the analysis of favorability in Experiment 2, “a” represents the background expression, with “a1” indicating a happy background expression, “a2” indicating a neutral background expression, and “a3” indicating a fearful background expression; “b” represents the micro-expression, with “b1” indicating a happy micro-expression, “b2” indicating a neutral micro-expression, and “b3” indicating a fearful micro-expression; “c” represents the mask, with “c1” indicating wearing a white opaque mask and “c2” indicating wearing a transparent mask. For example, if the *p*-value under “a1b1c1-a2b1c1” is significant, it means that the favorability of identifying happy micro-expressions under happy background expressions with a white opaque mask is significantly higher than that of identifying happy micro-expressions under neutral background expressions with a white opaque mask. “M1–M2” represents the difference in means between two conditions. “SE” stands for the standard error. *p* < 0.05 stands for significant.

Judgment Mode	M1–M2	SE	*p*
a1b1c1-a2b1c1	0.484	0.106	<0.001
a1b1c1-a3b1c1	0.654	0.135	<0.001
a2b1c1-a3b1c1	0.170	0.065	0.013
a1b1c2-a2b1c2	0.482	0.121	<0.001
a1b1c2-a3b1c2	0.717	0.136	<0.001
a2b1c2-a3b1c2	0.235	0.063	0.001
a2b2c1-a1b2c1	0.240	0.096	0.017
a2b2c2-a1b2c2	0.229	0.084	0.010
a2b2c2-a3b2c2	0.284	0.111	0.015
a1b3c2-a3b3c2	0.335	0.111	0.004
a2b3c2-a3b3c2	0.323	0.084	<0.001
a3b2c1-a1b2c1	0.045	0.125	0.720
a2b2c1-a3b2c1	0.195	0.101	0.061
a1b2c2-a3b2c1	0.055	0.130	0.676
a1b3c1-a3b3c1	0.131	0.088	0.143
a1b3c1-a2b3c1	0.129	0.065	0.053
a2b3c1-a3b3c1	0.002	0.078	0.975
a1b3c2-a2b3c2	0.012	0.097	0.898

**Table 8:** In the analysis of favorability in Experiment 2, “a” represents the background expression, with “a1” indicating a happy background expression, “a2” indicating a neutral background expression, and “a3” indicating a fearful background expression; “b” represents the micro-expression, with “b1” indicating a happy micro-expression, “b2” indicating a neutral micro-expression, and “b3” indicating a fearful micro-expression; “c” represents the mask, with “c1” indicating wearing a white mask and “c2” indicating wearing a transparent mask. For example, if the *p*-value under “a1b1c2-a1b1c1” is significant, it means that the favorability of identifying happy micro-expressions under happy background expressions with a transparent mask is significantly higher than that of identifying happy micro-expressions under happy background expressions with a white mask. “M1–M2” represents the difference in means between two conditions. “SE” stands for the standard error. *p* < 0.05 stands for significant.

Judgment Mode	M1–M2	SE	*p*
a1b1c2-a1b1c1	0.400	0.078	<0.001
a2b1c2-a2b1c1	0.401	0.087	<0.001
a3b1c2-a3b1c1	0.336	0.097	0.001
a3b3c1-a3b3c2	0.264	0.059	<0.001
a1b2c2-a1b2c1	0.009	0.070	0.901
a1b3c1-a1b3c2	0.060	0.078	0.449
a2b2c1-a2b2c2	0.003	0.063	0.968
a2b3c2-a2b3c1	0.056	0.059	0.348
a3b2c1-a3b2c2	0.091	0.063	0.154

**Table 9:** In the analysis of accuracy (ACC) in Experiment 3, “a” represents the background expression, with “a1” indicating a happy background expression, “a2” indicating a neutral background expression, and “a3” indicating a fearful background expression; “b” represents the micro-expression, with “b1” indicating a happy micro-expression, “b2” indicating a neutral micro-expression, and “b3” indicating a fearful micro-expression; “c” represents the mask, with “c1” indicating wearing a white mask and “c2” indicating wearing a patterned mask. For example, if the *p*-value under “a1b1c1-a3b1c1” is significant, it means that the accuracy of identifying happy micro-expressions under happy background expressions with a white mask is significantly higher than that of identifying happy micro-expressions under fearful background expressions with a white mask. “M1–M2” represents the difference in means between two conditions. “SE” stands for the standard error. *p* < 0.05 stands for significant.

Judgment Mode	M1–M2	SE	*p*
a1b1c1-a3b1c1	0.166	0.034	0.000
a2b1c1-a3b1c1	0.101	0.027	0.001
a1b1c2-a2b1c2	0.085	0.035	0.019
a1b1c2-a3b1c2	0.137	0.033	0.000
a2b2c1-a1b2c1	0.340	0.039	0.000
a2b2c1-a3b2c1	0.274	0.039	0.000
a2b2c2-a1b2c2	0.353	0.035	0.000
a2b2c2-a3b2c2	0.264	0.043	0.000
a3b2c2-a1b2c2	0.089	0.037	0.020
a1b1c1-a2b1c1	0.065	0.036	0.077
a2b1c2-a3b1c2	0.053	0.032	0.111
a3b2c1-a1b2c1	0.066	0.037	0.078
a1b3c1-a2b3c1	0.016	0.021	0.450
a3b3c1-a1b3c1	0.030	0.036	0.415
a3b3c1-a2b3c1	0.046	0.033	0.169
a1b3c2-a2b3c2	0.029	0.021	0.179
a3b3c2-a1b3c2	0.010	0.030	0.744
a3b3c2-a3b3c2	0.039	0.028	0.168

**Table 10:** In the analysis of accuracy (ACC) in Experiment 3, “a” represents the background expression, with “a1” indicating a happy background expression, “a2” indicating a neutral background expression, and “a3” indicating a fearful background expression; “b” represents the micro-expression, with “b1” indicating a happy micro-expression, “b2” indicating a neutral micro-expression, and “b3” indicating a fearful micro-expression; “c” represents the mask, with “c1” indicating wearing a white mask and “c2” indicating wearing a patterned mask. For example, if the *p*-value under “a2b1c1-a2b1c2” is significant, it means that the accuracy of identifying happy micro-expressions under neutral background expressions with a white mask is significantly higher than that of identifying happy micro-expressions under neutral background expressions with a patterned mask. “M1–M2” represents the difference in means between two conditions. “SE” stands for the standard error. *p* < 0.05 stands for significant.

Judgment Mode	M1–M2	SE	*p*
a2b1c1-a2b1c2	0.046	0.018	0.012
a1b1c1-a1b1c2	0.026	0.015	0.090
a1b2c1-a1b2c2	0.011	0.023	0.623
a1b3c2-a1b3c1	0.028	0.017	0.104
a2b2c2-a2b2c1	0.001	0.007	0.850
a2b3c2-a2b3c1	0.015	0.019	0.444
a3b1c2-a3b1c1	0.002	0.027	0.928
a3b2c2-a3b2c1	0.011	0.021	0.594
a3b3c2-a3b3c1	0.008	0.019	0.692

**Table 11:** In the analysis of reaction time (RT) in Experiment 3, “a” represents the background expression, with “a1” indicating a happy background expression, “a2” indicating a neutral background expression, and “a3” indicating a fearful background expression; “b” represents the micro-expression, with “b1” indicating a happy micro-expression, “b2” indicating a neutral micro-expression, and “b3” indicating a fearful micro-expression; “c” represents the mask, with “c1” indicating wearing a white mask and “c2” indicating wearing a patterned mask. For example, if the *p*-value under “a3b3c1-a1b3c1” is significant, it means that the reaction time for identifying fearful micro-expressions under fearful background expressions with a white mask is significantly longer than that for identifying fearful micro-expressions under happy background expressions with a white mask. “M1–M2” represents the difference in means between two conditions. “SE” stands for the standard error. *p* < 0.05 stands for significant.

Judgment Mode	M1–M2	SE	*p*
a3b3c1-a1b3c1	217.075	53.207	<0.001
a3b3c1-a2b3c1	230.104	58.193	<0.001
a3b3c2-a1b3c2	270.877	40.249	<0.001
a3b3c2-a2b3c2	178.809	46.728	<0.001
a1b1c1-a2b1c1	109.861	57.383	0.063
a1b1c1-a3b1c1	25.798	56.118	0.648
a3b1c1-a2b1c1	84.064	63.026	0.190
a1b1c2-a2b1c2	8.381	63.569	0.896
a1b1c2-a3b1c2	42.706	54.066	0.434
a2b1c2-a3b1c2	34.325	59.356	0.566
a1b2c1-a2b2c1	7.021	66.934	0.917
a1b2c1-a3b2c1	10.690	59.913	0.859
a2b2c1-a3b2c1	3.669	54.493	0.947
a1b2c2-a2b2c2	41.570	75.170	0.583
a1b2c2-a3b2c2	101.379	73.757	0.177
a2b2c2-a3b2c2	59.809	53.523	0.271
a1b3c1-a2b3c1	13.029	60.998	0.832
a2b3c2-a1b3c2	92.069	53.137	0.091

**Table 12:** In the analysis of favorability in Experiment 3, “a” represents the background expression, with “a1” indicating a happy background expression, “a2” indicating a neutral background expression, and “a3” indicating a fearful background expression; “b” represents the micro-expression, with “b1” indicating a happy micro-expression, “b2” indicating a neutral micro-expression, and “b3” indicating a fearful micro-expression; “c” represents the type of mask, with “c1” indicating wearing a white mask and “c2” indicating wearing a patterned mask. For example, if the *p*-value under “a1b1c1-a2b1c1” is significant, it means that the favorability of identifying happy micro-expressions under happy background expressions with a white mask is significantly higher than that of identifying happy micro-expressions under neutral background expressions with a white mask. “M1–M2” represents the difference in means between two conditions. “SE” stands for the standard error. *p* < 0.05 stands for significant.

Judgment Mode	M1–M2	SE	*p*
a1b1c1-a2b1c1	0.301	0.085	0.001
a1b1c1-a3b1c1	0.720	0.114	<0.001
a2b1c1-a3b1c1	0.419	0.076	<0.001
a1b1c2-a2b1c2	0.356	0.079	<0.001
a1b1c2-a3b1c2	0.535	0.111	<0.001
a2b1c2-a3b1c2	0.179	0.067	0.011
a2b2c1-a1b2c1	0.125	0.091	0.176
a2b2c1-a3b2c1	0.075	0.082	0.366
a3b2c1-a2b2c1	0.050	0.107	0.644
a1b2c2-a3b2c2	0.037	0.135	0.783
a2b2c2-a1b2c2	0.101	0.116	0.389
a2b2c2-a3b2c2	0.139	0.090	0.129
a1b3c1-a2b3c1	0.065	0.098	0.511
a1b3c1-a3b3c1	0.109	0.116	0.352
a2b3c1-a3b3c1	0.044	0.077	0.574
a1b3c2-a2b3c2	0.026	0.068	0.703
a1b3c2-a3b3c2	0.114	0.072	0.122
a2b3c2-a3b3c2	0.088	0.080	0.281

**Table 13:** In the analysis of favorability in Experiment 3, “a” denotes the background expression, with “a1” representing a happy background expression, “a2” a neutral background expression, and “a3” a fearful background expression; “b” denotes the micro-expression, with “b1” representing a happy micro-expression, “b2” a neutral micro-expression, and “b3” a fearful micro-expression; “c” denotes the type of mask, with “c1” representing wearing a white mask and “c2” wearing a patterned mask. For instance, if the *p*-value under “a1b1c1-a2b1c1” is significant, it indicates that the favorability of identifying happy micro-expressions under happy background expressions with a white mask is significantly higher than that of happy micro-expressions under neutral background expressions with a white mask. “M1–M2” represents the difference in means between two conditions. “SE” stands for the standard error. *p* < 0.05 stands for significant.

Judgment Mode	M1–M2	SE	*p*
a2b1c1-a2b1c2	0.117	0.048	0.018
a3b1c2-a3b1c1	0.122	0.050	0.020
a1b1c1-a1b1c2	0.062	0.057	0.282
a1b2c2-a1b2c1	0.006	0.063	0.921
a1b3c1-a1b3c2	0.060	0.077	0.442
a2b2c1-a2b2c2	0.017	0.039	0.656
a2b3c1-a2b3c2	0.021	0.053	0.693
a3b2c1-a3b2c2	0.081	0.040	0.050
a3b3c1-a3b3c2	0.065	0.044	0.145

## Data Availability

The datasets used and/or analyzed during the current study are available from the corresponding author (psyclzhu@yzu.edu.cn) on reasonable request.
